# Rapid reshaping of the soil microbiome and metabolome during short-term flooding and draining in rice

**DOI:** 10.3389/fmicb.2025.1632744

**Published:** 2025-09-02

**Authors:** Yanyao Lu, Donghong Lai, Shuo Cai, Haiyuan Wang, Zejun Hu, Qiangqiang Xiong

**Affiliations:** ^1^Jiangsu Key Laboratory of Crop Genetics and Physiology/Jiangsu Key Laboratory of Crop Cultivation and Physiology, Agricultural College of Yangzhou University, Yangzhou, China; ^2^Jiangxi Irrigation Experiment Central Station, Nanchang, China; ^3^Key Laboratory of Germplasm Innovation and Genetic Improvement of Grain and Oil Crops (Co-construction by Ministry and Province), Ministry of Agriculture and Rural Affairs, Crop Breeding and Cultivation Research Institute, Shanghai Academy of Agricultural Sciences, Shanghai, China; ^4^Jiangsu Co-Innovation Center for Modern Production Technology of Grain Crops, Yangzhou University, Yangzhou, China

**Keywords:** flooding, rice, soil microorganisms, metabolomics, lipidomics

## Abstract

The impact of the flooding-draining process on soil ecosystems is complex and dynamic. However, the specific effects of different drainage durations on soil microorganisms and metabolites remain unclear. This study adopted a multi-omics research method. After nontargeted metabolomics analysis of lipids as the main metabolite, microbial diversity analysis and lipidomics analysis were conducted to determine the main influencing factors. Subsequently, correlation analysis was performed with physiological and biochemical data to logically explore the changes in soil microorganisms and metabolites during the drainage process (Day 1 after drainage, R1; Day 2, R2; Day 3, R3; Day 4, R4; and Day 5, R5). The results revealed that S-PPO, S-POD, and S-CAT decreased with prolonged drainage time, whereas the soil redox potential (Eh-mV) and POD increased. Among the various postdrainage comparison groups, lipids and lipid-like molecules were the predominant metabolites. Among lipids, the TG subclass of glycerolipids (GLs) and the Cer subclass of sphingolipids (SPs) were the most abundant. The TG subclass was consistently present in the lipid correlation networks across all comparison groups, with TG (15:0/18:1/18:1) exhibiting significant differences between the R4 and R1 groups. Redox reactions involving lipids were associated mainly with triglycerides, with the most pronounced reduction observed on the second day postdrainage. The most pronounced lipid reduction reaction was observed on the second day after drainage. Notable differences in bacterial abundance were detected between the R4 and R5 groups. At the phylum level, the dominant bacterial communities primarily comprised Actinobacteriota and Chloroflexi, with the bacterial community structure being significantly influenced by drainage. The predominant fungal communities were composed of mainly Ascomycota and Rozellomycota. Actinobacteriota and triglyceride (TG) lipids were the major components affected during the drainage period. Correlations were identified among environmental factors, lipids, and microbial communities, indicating their cooperative interactions. The results of this study indicate that with the increase in water intake time, the redox reactions in soil lipids and the richness of bacterial communities in rice soil significantly increase. At the same time, rapid remodeling can have an impact on soil ecosystems, which helps to better understand the adaptation strategies of rice soil ecosystems under adversity.

## Introduction

The increasing severity of global climate change has intensified extreme weather events, posing significant challenges to global ecosystems (Bolan et al., 2023). Flooding, as a typical manifestation of this phenomenon, has profoundly impacted agricultural production and the ecological environment while also significantly disturbing soil microbial ecosystems (Han et al., 2024; Furtak and Wolińska, 2023).

Soil microorganisms are the most active and sensitive biological components of the soil ecosystem. They are essential for sustaining soil fertility and boosting crop yields by participating in the decomposition, transformation, and energy flow of organic matter (Jansson and Hofmockel, 2020). However, flooding disasters can lead to decreased soil aeration and reduced redox potential, which affect the composition and function of soil microbial communities (Ponting et al., 2021). Previous studies have extensively investigated the effects of soil drying and rewetting on microbial communities, metabolites, proteins, and gene expression (Weitz et al., 2020; Couvillion et al., 2023; Warren, 2020). However, our understanding of how flooded paddy soils influence microbial physiology and metabolism following water withdrawal remains limited. Microbial communities are highly sensitive to environmental disturbances, which can simultaneously reshape both their metabolic activities and taxonomic structure. Understanding how individual microbial populations adjust their metabolic functions under stress conditions is essential, as such adaptations influence interspecies metabolic networks and cooperative interactions. These shifts at the metabolic level may ultimately drive broader changes in community assembly and impact ecosystem-level processes such as nutrient cycling and energy flow (Zelezniak et al., 2015). Additionally, soil microorganisms can produce a range of biomarkers, such as lipids, through their metabolic activities. These biomarkers can indicate past conditions in soil microbial communities, providing important clues for understanding the response mechanisms of soil microorganisms during the flooding–drainage process (Summons et al., 2022). As a key component of soil microbial communities, the lipidome can reflect the diversity and functionality of these communities according to its composition and variations.

The rapid reshaping of soil microbial communities and lipidomes during the flooding–drainage process may be influenced by various factors, including soil physicochemical properties, the initial state of the microbial communities, and the duration of flooding (Vives-Peris et al., 2020). Different types of microorganisms respond to environmental changes at varying speeds and in different ways. For example, some anaerobic microorganisms can quickly adapt to hypoxic environments, whereas some aerobic microorganisms may be suppressed during the initial stages of flooding but can rapidly recover after drainage (Yuan et al., 2023). The soil organic matter and nutrient contents can also affect microbial metabolic activities and community structure (Ling et al., 2021). Nontargeted metabolomics can detect all detectable metabolites in soil samples in an unbiased manner, thereby revealing extensive changes in soil metabolic pathways during the flooding–drainage process. These metabolites, as direct products of microbial activity or response markers to environmental stress, can provide detailed information about changes in the functional status of soil ecosystems (RoyChowdhury et al., 2022). Soil microorganisms can cause carbon fixation in the soil during the remodeling process (Afridi et al., 2022). Meanwhile, the utilization of soil may lead to changes in soil microbial diversity, thereby affecting greenhouse gas emissions (Kroeger et al., 2021). Nutrient availability can also be altered by changes in microorganisms and lipids (Pedrinho et al., 2024). Currently, omics technologies are becoming increasingly important in the field of rice research, and increasing numbers of studies are using omics research methods. Through the combination of multiple omics fields, scientific research can be conducted logically (Iqbal et al., 2021). Through nontargeted metabolomic analysis, we can identify the activation or inhibition of key metabolic pathways during the flooding–drainage process and thus enhance our understanding of the adaptive strategies of soil ecosystems under stress (Gui et al., 2024). Recent advances in mass spectrometry have significantly enhanced the fields of lipidomics and metabolomics, enabling the characterization and quantification of tens of thousands of features representing known lipid and metabolite species, far beyond the limited scope of traditional targeted analyses (Edwards, 2023). As an emerging research tool, lipidomics can provide detailed information about microbial membrane lipid composition and metabolic status and therefore help reveal microbial adaptive mechanisms during environmental changes. Moreover, compared to transcriptomic approaches that measure mRNA levels, lipid-based profiling provides a more temporally stable reflection of microbial metabolic states, capturing metabolic changes over longer timescales (Balser et al., 2019; Ding et al., 2021).

This research examines the dynamic alterations in soil microbial community structure, functionality, and metabolome throughout the flooding–drainage cycle. At present, the results obtained via conventional analysis are not accurate. However, multi-omics analysis can obtain final results through precise analysis at various omics levels (Shahrajabian and Sun, 2023). By integrating nontargeted metabolomics, lipidomics, and microbiome techniques, this study first analyzed the metabolite composition and major metabolites in the R1–R5 treatment group. Subsequently, the lipid metabolites and microorganisms in the R1–R5 treatment group were analyzed to further confirm the main lipid metabolites and microbial communities that affect the water return process and to perform correlation analysis with microbial and physiological biochemical data to determine the key factors in the process of flooding and water return. Additionally, we explore the factors driving changes in soil microorganisms and lipidomes by combining measurements of soil physicochemical properties with environmental factor monitoring. This study aims to elucidate the rapid remodeling mechanisms of the soil microbiota and lipidomes during the flooding–water recession process. By integrating soil microbial ecology with lipidomics, our findings contribute to the theoretical framework of soil ecology and provide a scientific basis and practical guidance for ecological conservation and sustainable agricultural development. Due to the rapid remodeling of microorganisms and lipids in soil under stress, which can have an impact on soil ecosystem functions, this integrated method help solve soil ecosystem problems. Moreover, this research offers new perspectives and strategies for responding to extreme climatic events under the context of climate change.

## Materials and methods

### Experimental materials and growth conditions

The experiment was conducted in 2023 at the College of Agriculture, Yangzhou University (the specific coordinates are 119.423282 degrees east longitude and 32.389204 degrees north latitude), using Yangzi 6, an early-maturing late japonica conventional rice variety, as the experimental material in a pot cultivation setup.

The pot cultivation method was adopted following the approach of Zhu et al. (2022). Field management was standardized before transplantation, and after transplantation, irrigation and pest control were carried out according to high-yield cultivation practices.

The experiment was conducted using plastic buckets (inner diameter: 25 cm, height: 30 cm) for the bucket cultivation. The seeds were sown on May 26, 2023, and rice plants were transplanted on June 18. Cultivated soil was collected from the 0–20 cm plow layer of paddy fields, air-dried naturally, and then ground to pass through a 100-mesh sieve. Seedlings with good and consistent growth were selected for transplanting, with 3 holes per barrel, 2 seedlings per hole, and 15 kg soil per pot. Post-transplantation water, disease, and pest management followed high-yield cultivation practices. The plants were placed in two large water tanks, water was added, and the barrels were submerged to the upper part of the rice seedlings. The plants remained submerged for 6 days. Samples were collected after 6 days of flooding (0 days, soil after flooding), and on the 1st, 2nd, 3rd, 4th, and 5th days after rewetting, corresponding to R1, R2, R3, R4, and R5, respectively.

### Sample collection

Soil rhizosphere microorganisms and root samples were collected following the method outlined by Sun et al. (2024).

Rice rhizosphere soil samples were collected 10 cm from the plant at a depth of 20 cm and placed in sampling tubes, with 10 g of soil obtained per treatment.

For rice root system sampling, whole rice plants were carefully excavated using appropriate tools, and the rhizosphere soil adhering to the roots was collected.

Three biological replicates of both the rhizosphere soil and the roots were obtained for each treatment. All samples were flash-frozen in liquid nitrogen prior to transport to the laboratory and subsequently stored at −80 °C in an ultra-low-temperature freezer until analysis.

### Metabolite extraction, detection, and data analysis

Metabolite extraction and detection were conducted following the method described by Zhu et al. (2024). Soil samples (50.00 g) were accurately weighed for the extraction of metabolites using 400 μL of a methanol–water (4:1, v/v) solution with 0.02 mg mL^−1^ L-2-chlorophenylalanin as the internal standard. A pooled quality control (QC) sample was created by combining equal volumes of all samples as part of the system conditioning and quality control procedures.

Metabolite data analysis was performed following the method described by Xiong et al. (2022). LC-MS analysis was performed using the Thermo Fisher Scientific UHPLC-Q Exactive platform.

Chromatographic separation was achieved on a UPLC HSS T3 column (100 mm × 2.1 mm, 1.8 μm) with the following parameters: column temperature maintained at 40 °C, mobile phase consisting of (A) 0.1% formic acid aqueous solution and (B) 0.1% formic acid in propanol/acetonitrile mixture, and a constant flow rate of 0.4 mL/min. Sample injection volume was set at 2 μL. Mass spectrometric detection employed an electrospray ionization (ESI) source with dual polarity scanning capability (positive/negative ion modes).

### Microbial DNA extraction and amplicon analysis

DNA extraction and 16S sequencing were performed according to the method of Zhu et al. (2022). Microbial genomic DNA was extracted from the soil samples using the E.Z.N.A.^®^ Soil DNA Kit (Omega Bio-tek, Norcross, Georgia, United States). The V3–V4 hypervariable region of the bacterial 16S rRNA gene was amplified using the forward primer 338F (5′-ACTCCTACGGGAGGCAGCAG-3′) and the reverse primer 806R (5′-GGACTACHVGGGTWTCTAAT-3′). For fungal community, the ITS sequences were amplified using the primers ITS1F (5′-CTTGGTCATTTAGAGGAAGTAA-3′) and ITS4R (5′-TCCTCCGCTTATTGATATGC-3′). Primers were tailed with PacBio barcode sequences to distinguish each sample. Amplification reactions (20-μL volume) consisted of 4 μL of 5 × FastPfu buffer, 2 μL of 2.5 mM dNTPs, 0.8 μL of forward primer (5 μM), 0.8 μL of reverse primer (5 μM), 0.4 μL of FastPfu DNA Polymerase, 10 ng of template DNA and DNase-free water. PCR amplification was performed as follows: initial denaturation at 95 °C for 3 min; 27 cycles of denaturing at 95 °C for 30 s, annealing at 55 °C for 30 s and extension at 72 °C for 45 s; single extension at 72 °C for 10 min; and then holding at 4 °C (T100 Thermal Cycler PCR thermocycler, BIO-RAD, United States). After electrophoresis, the PCR products were purified using AMPure^®^ PB beads (Pacific Biosciences, CA, United States) and quantified with Qubit 4.0 (Thermo Fisher Scientific, United States). Sequencing of the equimolar pooled purified amplicons was performed using paired-end sequencing on the Illumina PE250 platform (Illumina, San Diego, United States).

Equimolar pooled purified amplicons were sequenced using paired-end sequencing on an Illumina PE250 platform (Illumina, San Diego, United States). Post-demultiplexing, sequences underwent quality filtering using fastp (v0.19.6) and were subsequently merged with FLASH (v1.2.11). High-quality sequences were de-noised using the DADA2 plugin in the QIIME2 (version 2020.2) pipeline with recommended parameters, achieving single nucleotide resolution based on sample error profiles. Amplicon sequence variants (ASVs) are typically referred to as DADA2-denoised sequences. ASVs were taxonomically assigned using the Naive Bayes consensus taxonomy classifier in QIIME2 with the SILVA 16S rRNA database (v138). Metagenomic functions were predicted using PICRUSt2, based on ASV representative sequences. PICRUSt2 is a software suite that utilizes HMMER to align ASV representative sequences with reference sequences. ASV representative sequences were integrated into a reference tree using EPA-NG and Gappa. The 16S gene copies were normalized to the levels of castor. MinPath was employed to predict gene family profiles and map them to gene pathways. The entire analysis process was aligned with PICRUSt2 protocols.

### Metabolomics analysis

Raw LC-MS data were processed using Progenesis QI software (Waters Corporation, Milford, United States), and a three-dimensional data matrix in CSV format was generated, containing sample metadata, metabolite identities, and mass spectral intensities. Internal standards and known false-positive peaks—including instrumental noise, column bleed, and derivatization artifacts—were removed, followed by deredundancy and peak pooling. Metabolites were identified by matching against the HMDB,^1^ Metlin,^2^ and the self-curated Majorbio Database (MJDB) from Majorbio Biotechnology Co., Ltd. (Shanghai, China).

The annotated data matrix was then uploaded to the Majorbio Cloud Platform^3^ for statistical and bioinformatics analyses. Variables with more than 80% non-zero values in at least one group were retained to reduce missingness. Remaining missing values were imputed using the minimum observed intensity. To correct for systematic errors introduced by sample preparation or instrument variation, total sum normalization was applied. Variables with a relative standard deviation (RSD) exceeding 30% in quality control (QC) samples were excluded. The data were subsequently log10-transformed to approximate a normal distribution.

Multivariate statistical analyses, including principal component analysis (PCA) and orthogonal partial least squares discriminant analysis (OPLS-DA), were conducted using the ropls package (Version 1.6.2) in R. Model stability was validated by 7-fold cross-validation. Differential metabolites were identified based on variable importance in projection (VIP) scores greater than 1 from the OPLS-DA model and *p*-values less than 0.05 from Student’s t tests. Functional interpretation of differential metabolites was performed through pathway annotation using the KEGG database.^4^ Enrichment analysis was carried out using the scipy.stats module in Python, with Fisher’s exact test used to identify pathways significantly associated with experimental treatments.

### Data preprocessing and annotation

Data preprocessing refers to the method of Zhu et al. (2022). After MS detection, the raw LC-MS data were preprocessed and annotated. Data processing was performed using Progenesis QI software (Waters Corporation, Milford, United States). The output was a CSV-formatted 3D data matrix containing sample details, metabolite identifiers, and MS response intensities.

To enhance data accuracy, the matrix was refined by removing internal standard peaks and known false-positive peaks (e.g., noise, column bleed, and derivatization reagent peaks). The matrix was further processed by deduplication and peak merging. Metabolites were identified using databases such as HMDB, Metlin, and MajorBio.

The processed data were then imported into the MajorBio cloud platform for analysis. Metabolic features present in at least 80% of the sample groups were retained. For samples with metabolite levels below the quantification threshold, the minimum value was estimated. Variables with a relative standard deviation (RSD) exceeding 30% in QC samples were excluded. A final data matrix was created using log10 transformation to facilitate further analysis.

### Data analysis

Physiological and biochemical data were organized and visualized using Adobe Illustrator CS6 and WPS 2021. Correlation analysis and significance testing were performed using SPSS 18.0, with statistical significance determined by Tukey’s test.

## Results

### Analysis of physiological and biochemical indicators

**Figure 1:**
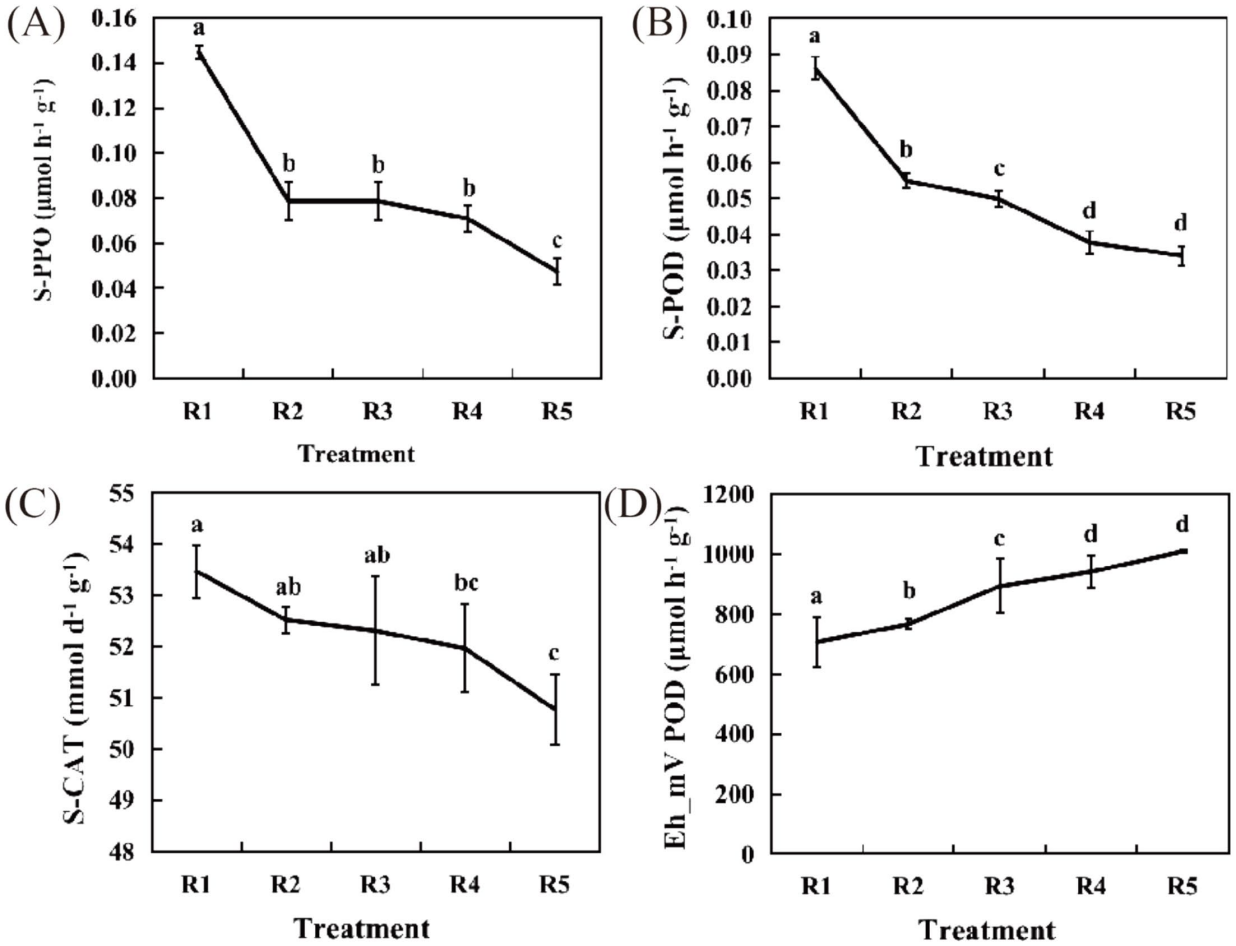
Physiological and biochemical indicators. **(A)** Soil polyphenol oxidase (S-PPO) activity. **(B)** Soil peroxidase (S-POD) activity. **(C)** Soil catalase (S-CAT) activity. **(D)** Redox potential. The short lines in the line graphs represent standard error. The data are shown as the mean ± s.e.m. (*n* = 3). Lowercase letters indicate significance at the 0.05 level.

The PPO activity of R3 was consistent with that of R2 and both were significantly lower than that of R1 (*p* < 0.05), showing no significant difference, whereas R4 exhibited a 50.93% decrease compared with R1, and R5 presented a substantial decline of 67.26% relative to R1 (Figure 1A). POD activity also showed a continuous decrease from R1 to R5, with significant variations observed between R1 and R2 (*p* < 0.05), as well as between R3 and R4, R5. Specifically, R2 was significantly lower than R1 (*p* < 0.05), with a reduction of 36.3%, while R3 displayed a decline of 42.04% compared with R1. The POD activities in R4 and R5 decreased by 56.21 and 60.39%, respectively, relative to those in R3 (Figure 1B).

CAT activity followed a similar trend to both PPO and POD activity, exhibiting a gradual decline. Notable differences were observed between R1 and R4, as well as between R1 and R5. Compared with R1, R2 showed a minor reduction of 1.8%, while R3 decreased by 2.16%. The CAT activity in R4 decreased by 2.81% compared to that in R1, and R5 exhibited the most significant reduction (*p* < 0.05), with a 5.03% decline relative to that in R1 (Figure 1C).

The redox potential (Eh) reflects the aeration status of soil. As the duration of drainage increased, Eh progressively rose, remaining positive, indicating that the sampled soil was in a reduced state. Significant differences were evident among R1, R2, R3, R4, and R5 (*p* < 0.05). From R2 onward, Eh showed a slight increase in each treatment, with R2 increasing by an average of 60 mV compared with R1, representing a 7.83% increase; R3 increased by 187 mV compared with R1, or 20.84%; R4 increased by 235 mV compared with R1, or 24.97%; and R5 increased by 382 mV compared with R1, or 37.89% (Figure 1D). This suggests that as the processes of flooding and drainage progress, soil physiological and biochemical activities gradually decline, accompanied by an increase in redox potential. We hypothesize that these changes in soil physiological and biochemical processes may be associated with redox transformations involving soil microbial communities and lipid components.

### Metabolic profiling analysis

**Figure 2:**
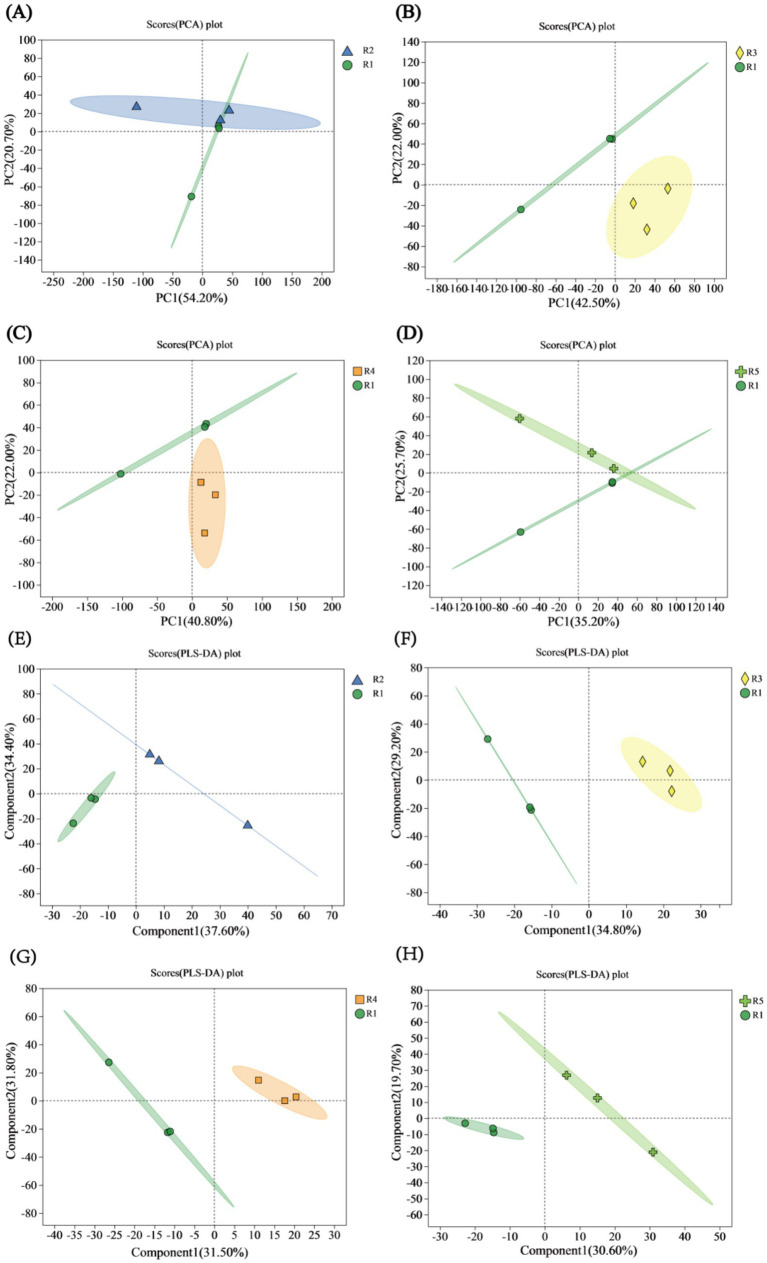
PCA and PLS-DA score plots. **(A)** PCA score plot for the R2 vs. R1 comparison. **(B)** PCA score plot for the R3 vs. R1 comparison. **(C)** PCA score plot for the R4 vs. R1 comparison. **(D)** PCA score plot for the R5 vs. R1 comparison. **(E)** PLS-DA score plot for the R2 vs. R1 comparison. **(F)** PLS-DA score plot for the R3 vs. R1 comparison. **(G)** PLS-DA score plot for the R4 vs. R1 comparison. **(H)** PLS-DA score plot for the R5 vs. R1 comparison.

Principal component analysis (PCA) on the four comparison groups revealed that in the comparison between R2 and R1, PC1 accounted for 54.20%, and PC2 accounted for 20.70%, with some overlap suggesting a certain degree of similarity (Figure 2A). In the comparison between R3 and R1, PC1 accounted for 42.50% of the variation, and PC2 accounted for 22.00%, highlighting significant differences (*p* < 0.05), as the two groups were distinctly separated (Figure 2B). For the comparison between R4 and R1, PC1 had a value of 40.80%, and PC2 had a value of 22.00%, with no overlap and with notable differences observed (Figure 2C). In the comparison between R5 and R1, PC1 accounted for 35.20%, and PC2 accounted for 25.70%, indicating some overlap and similarity (Figure 2D).

Partial least squares discriminant analysis (PLS-DA) was performed on the metabolite profiles of the four comparison groups. In the comparison between R2 and R1, the explained variances of Component 1 (Comp1) and Component 2 (Comp2) were 37.60 and 34.40%, respectively, with a greater intergroup separation observed in the R2 group compared to the N1 treatment group (Figure 2E). In the comparison between R3 and R1, Comp1 and Comp2 accounted for 34.80 and 25.70% of the variance, respectively. For the R4 vs. R1 comparison, Comp1 and Comp2 explained 31.50 and 31.80% of the variance, respectively. Notably, both R3 and R4 exhibited lower intergroup separation compared to the R1 treatment group (Figures 2F,G). In the R5 vs. R1 comparison, Comp1 and Comp2 accounted for 30.60 and 19.70% of the variance, respectively, with the R5 group showing greater intergroup separation than the R1 treatment group (Figure 2H). Overall, all four comparison groups demonstrated clear and substantial intergroup separation, indicating strong classification performance and high reliability of the PLS-DA models.

### Nontargeted metabolomic analysis

**Figure 3:**
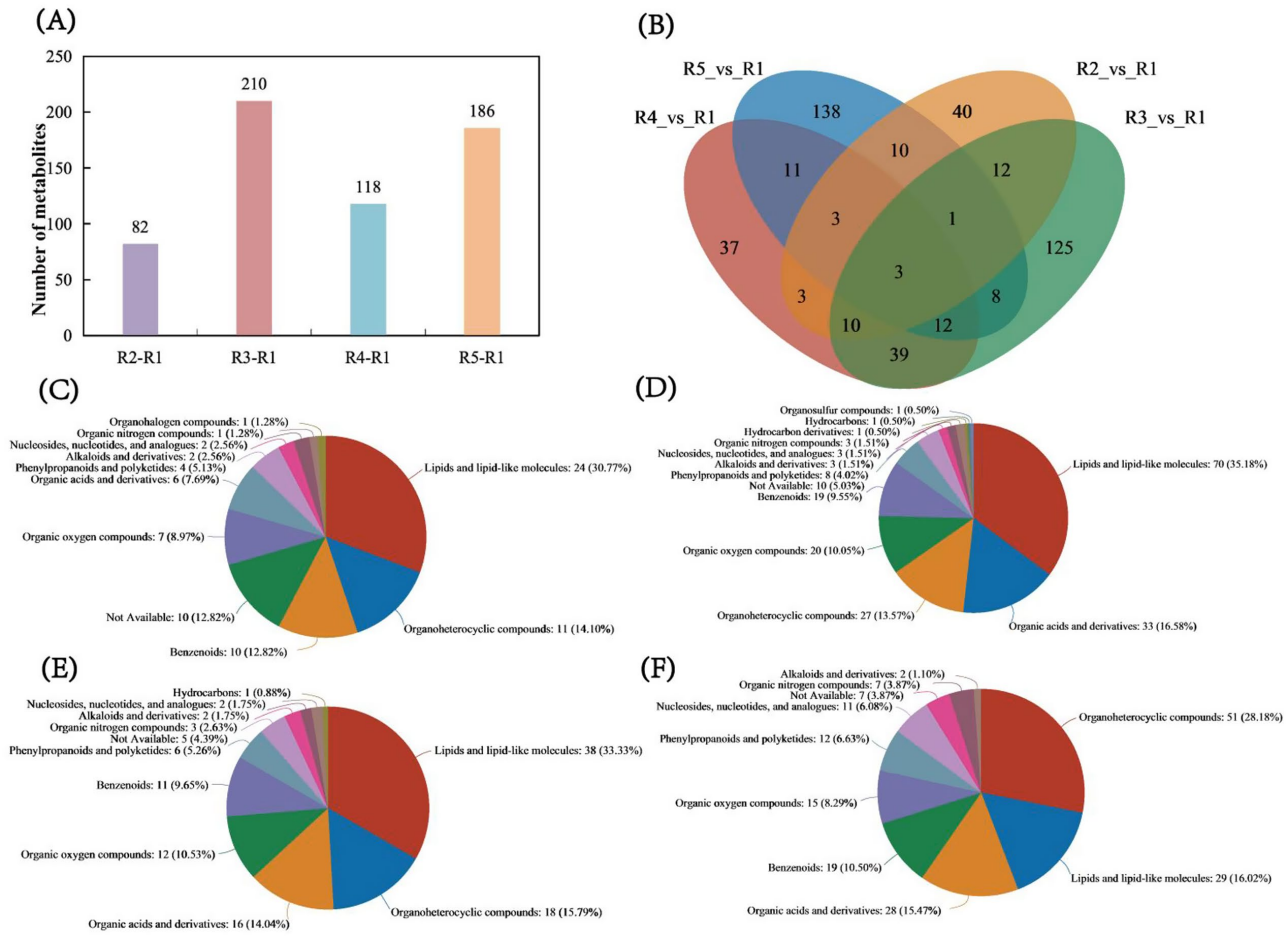
Analysis of metabolite numbers and shared metabolites. **(A)** Bar chart of metabolite numbers for each comparison group. **(B)** Venn diagram showing the number of shared metabolites among the comparison groups. Compound classification statistics. **(C)** Compound content statistics for the R2 vs. R1 comparison. **(D)** Compound content statistics for the R3 vs. R1 comparison. **(E)** Compound content statistics for the R4 vs. R1 comparison. **(F)** Compound content statistics for the R5 vs. R1 comparison. The selected HMDB hierarchy (superclass, class, or subclass) is displayed in descending order according to the number of metabolites, along with the percentage they occupy. In each pie chart, different colors represent different HMDB classifications, and the area reflects the relative proportion of metabolites within that classification.

From the bar chart of metabolite numbers across the comparison groups, the R2 vs. R1 comparison identified a total of 28 upregulated and 54 downregulated metabolites. In the R3 vs. R1 comparison, 45 upregulated and 165 downregulated metabolites were found, while in the R4 vs. R1 comparison, 118 differentially abundant metabolites were identified, comprising 32 upregulated and 86 downregulated metabolites. In the R5 vs. R1 comparison, 136 upregulated and 50 downregulated metabolites were identified (Figure 3A and Supplementary Tables S1–S4). The Venn diagram of metabolites for each comparison group shows that R2 vs. R1 has 40 unique metabolites, accounting for 48.78% of the total metabolites. In the R3 vs. R1 comparison, 125 unique metabolites were found, accounting for 59.52% of the total. For R4 vs. R1, 37 unique metabolites were identified, accounting for 31.36% of the total. In the R5 vs. R1 comparison, there were 138 unique metabolites, representing 74.19% of the total metabolites. Moreover, the number of metabolites common to all four groups was only 3, accounting for 0.005% of the total (Figure 3B). Analysis of the HMDB compound charts for the R2 vs. R1 comparison revealed the following composition: lipids and lipid-like molecules (30.77%), organic heterocyclic compounds (14.10%), benzoic acid derivatives (12.82%), organic oxidized compounds (8.97%), and organic acids and their derivatives (7.69%) (Figure 3C). In the R3 vs. R1 comparison, the composition was as follows: lipids and lipid-like molecules 35.18%, organic acids and derivatives 16.58%, organic heterocyclic compounds 13.57%, organic oxidized compounds 10.05%, and aromatic compounds, 9.55% (Figure 3D). In the R4 vs. R1 comparison, the composition was as follows: lipids and lipid-like molecules (33.33%), organic heterocyclic compounds (15.79%), organic acids and their derivatives (14.04%), organic oxidized compounds (10.53%), and benzoic acid derivatives (9.65%) (Figure 3E). In the comparison between R5 and R1, the composition was as follows: organic heterocyclic compounds constituted 28.18%, lipids and lipid-like molecules made up 16.02%, organic acids and their derivatives comprised 15.47%, benzoic acid derivatives accounted for 10.50%, and organic oxidized compounds represented 8.29% (Figure 3F).

### Distribution of lipid carbon chain length and unsaturation

**Figure 4:**
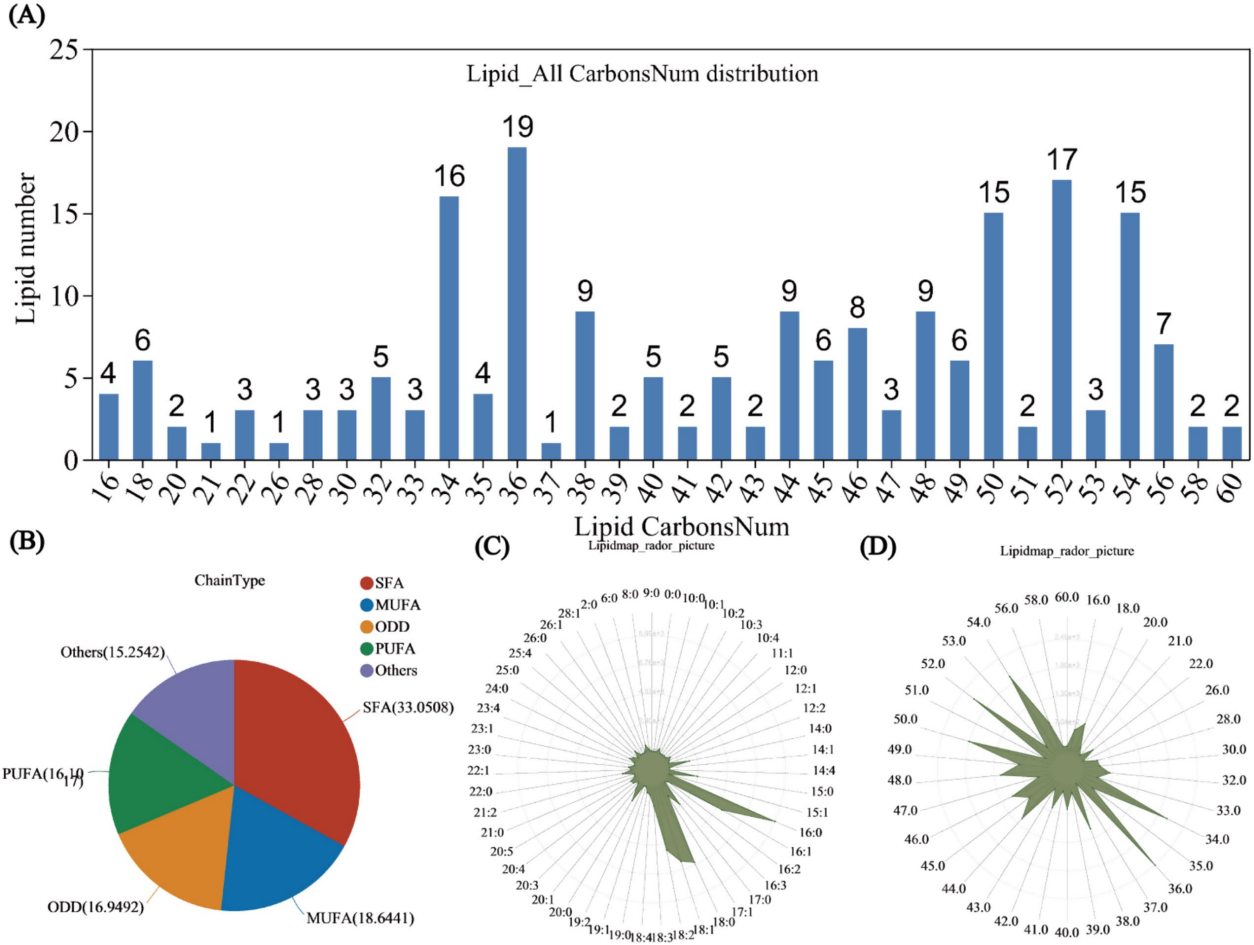
Lipid classification statistics. **(A)** Bar chart showing the distribution of lipid carbon chain lengths. The x-axis represents the lengths of lipid carbon chains, while the y-axis indicates the number of lipids with that carbon chain length. Lipid unsaturation classification statistics. **(B)** Pie chart illustrating the classification of lipid unsaturation. Different colors in the pie chart represent the quantities of various types, including saturated fatty acyls (SFA), monounsaturated fatty acyls (MUFA), polyunsaturated fatty acyls (PUFA), and odd-numbered fatty acyls (ODD). Lipid distribution content statistics. **(C)** Radar chart of lipid content by unsaturation type. **(D)** Radar chart of lipid content by carbon chain length. The grid lines, from inner to outer, represent lipid content from low to high, with the green shading formed by connecting the quantities classified by the number of double bonds.

The distribution bar chart of lipid metabolites categorized by carbon chain length revealed that the highest concentrations of lipid metabolites were observed at carbon chain lengths of 34, 36, 50, 52, and 54, with respective counts of 16, 19, 15, 17, and 15 species. Notably, lipid metabolites with a carbon chain length of 36 were the most prevalent, constituting 9.5% of the total lipid metabolites (Figure 4A). Additionally, 118 types of unsaturated lipids were identified. Saturated fatty acyls (SFAs) comprised 39 types, accounting for 33.0508% of the total; monounsaturated fatty acyls (MUFAs) consisted of 22 types, representing 18.6441%; polyunsaturated fatty acyls (PUFAs) included 19 types, making up 16.1017%; odd-chain fatty acyls (ODD) featured 20 types, accounting for 16.9492%; and the remaining unknown types numbered 18, comprising 15.2542% of the total (Figure 4B).

From the radar chart of lipid content by unsaturation type, the highest content was observed for 16:0 lipids, reaching 8,993. The next greatest contents were observed for 18:0 and 18:1 lipids, with contents of 6,580 and 6,129, respectively. The contents of 16:1 and 18:2 lipids were comparable, both with approximately 5,000, while 15:0 and 17:0 lipids exhibited similar contents of approximately 2,200 (Figure 4C). In the radar chart of lipid content by carbon chain length, the highest content was obtained for lipids with a carbon chain length of 36.0, followed by lipids with a length of 52.0, with contents of 2,493 and 2,208, respectively. The contents of lipids with a carbon chain length of 34.0 and 54.0 were similar (Figure 4D).

### Lipid classification analysis

**Figure 5:**
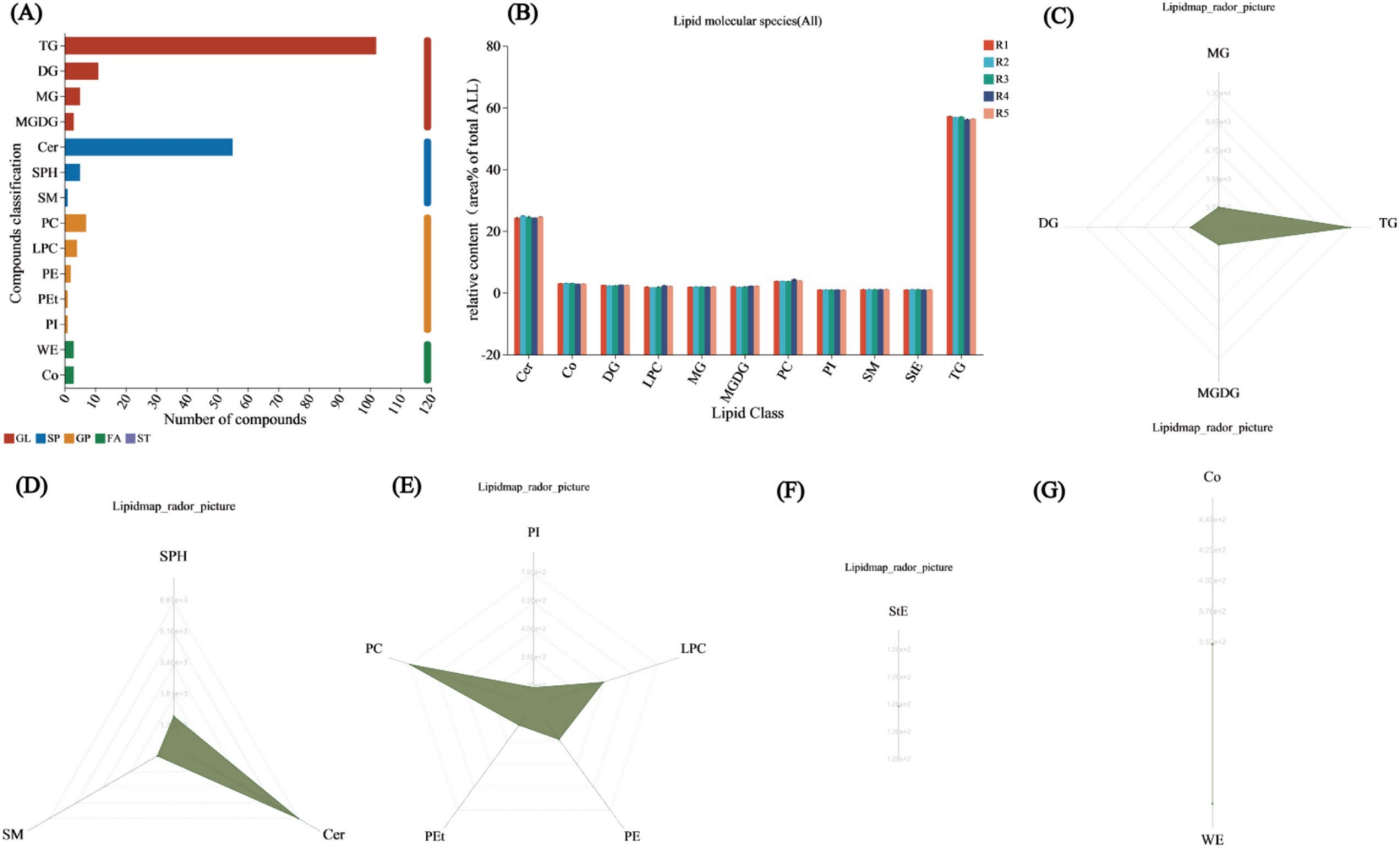
Lipid classification annotations. **(A)** Lipid classification statistics chart. The x-axis represents the number of identified lipids in each subclass, while the y-axis shows the names of the subclasses. Different colors denote the major classes to which each bar belongs, including fatty acyls (FA), glycerolipids (GL), glycerophospholipids (GP), sphingolipids (SP), sterol esters (ST), prenyl lipids (PR), glycolipids (SL), and polyketides (PK). Lipid classification analysis. **(B)** Lipid classification change statistics chart. The x-axis represents different lipid subclasses, and the y-axis shows the total content of different groups within the same lipid class. The black short bars represent error bars. Bars representing different groups are distinguished by different colors. **(C)** Radar chart of GL class content. **(D)** Radar chart of SP class content. **(E)** Radar chart of GP class content. **(F)** Radar chart of ST class content. **(G)** Radar chart of FA class content. The grid lines extend from inner to outer, representing lipid content from low to high, with the green shading formed by connecting the content of each classification.

In a total of five soil samples, 203 unique lipid species were identified. The lipids were categorized into three main classes: GLs, glycerophospholipids (GPs), and SPs. Among the unique lipids, 59.6% (121) were classified as GLs. These species were classified into subclasses of diacylglycerols (DGs), monostearin (MG), monogalactosyl diacylglycerols (MGDGs), and triacylglycerols (TGs). Among the 121 lipid species, 102 were classified into the TG subclass. Moreover, 15 (7.3%) unique glycerophospholipids were identified, including diacylglycerophosphocholine (PC), lysophosphatidylcholine (LPC), diacylglycerophosphoethanolamine (PE), phosphatidylethanolamine (PEt), and diacylglycerophosphoinositol (PI). Finally, 61 (30%) SPs were identified, including ceramides (Cer), SPH, and sphingomyelin (SM), with Cer being the most abundant, accounting for 90.1% of the identified SPs (Figure 5A). The lipid classification results indicate that TG represented the largest proportion among all lipid classes, averaging 56.73%, followed by Cer, with an average of 24.57%. The mean contents of the remaining nine lipid subclasses did not exceed 5%. The subclass with the lowest average content was PI, at 1.03% (Figure 5B). The radar chart of lipid content according to classification revealed that in the GL category, TG had the highest content, reaching 13,035, followed by DG, MG, and MGDG, with respective contents of 1,568, 685, and 371 (Figure 5C). In the SP radar chart, Cer was the most abundant, followed by sphingosine (Sph) and sphingomyelin (SM), with contents of 6,831, 730, and 130, respectively (Figure 5D). In the GP radar chart, PC had the highest content, reaching 793, followed by LPC > PE > PEt > PI, with contents of 452, 271, 167, and 119, respectively (Figure 5E). Only one subclass, StE, was found in the sterol (ST) radar chart, with a content of 126 (Figure 5F). Wax esters (WE) and cholesteryl esters (Co) were identified in the fatty acid (FA) radar chart, with contents of 446 and 352, respectively (Figure 5G).

### Analysis of lipid metabolism

This study examined the common and unique lipid metabolites across four comparison groups. The comparison of R2 and R1 revealed 41 lipid metabolites, 38 of which were upregulated and 3 of which were downregulated. For the R3 versus R1 comparison, 4 metabolites were upregulated, and 1 was downregulated. The R4 versus R1 comparison revealed 5 lipid metabolites, all of which were downregulated. In the R5 versus R1 comparison, there were 2 upregulated and 3 downregulated metabolites (Supplementary Figure S1A and Supplementary Tables S5–S8). To better understand the distribution of shared metabolites among these groups, a Venn diagram was generated, which indicated that R2 had 34 unique metabolites compared with R1, representing 82.92% of its total metabolites. The other three groups each had 2 unique metabolites, accounting for 40% of their total metabolites. Notably, there were no shared metabolites among the four groups (Supplementary Figure S1B).

### Differential lipid classification

To further identify the specific lipid types involved in the flooding-draining process, differential lipid classification analysis was conducted, and scatter plots were generated for the differential lipids across the four comparison groups. The scatter plot for R2 versus R1 revealed 41 differentially abundant lipids, categorized into TG, Cer, SM, PC, MG, and MGDG, with TG accounting for 18 metabolites, representing 43.90% of the total, followed by Cer with 16 metabolites, accounting for 39.02% (Supplementary Figure S2A). In the R3 versus R1 plot, 5 lipids were identified, including DG, PC, TG, and Cer lipids, such as TG:TG (15:0/16:1/16:1) and TG (18:0/16:0/18:3) (Supplementary Figure S2B). The R4 versus R1 plot revealed five lipids from the TG subclass: TG (16:0/16:0/16:0), TG (16:1/14:0/14:0), TG (15:0/18:1/18:1), TG (16:1/16:1/20:5), and TG (15:0/15:0/16:0) (Supplementary Figure S2C). In the comparison between R5 and R1, five lipids were identified, including TG, Cer, and MGDG, with three lipids classified under the TG subclass: TG (16:0/16:0/16:1), TG (15:0/18:1/18:1), and TG (16:1/18:3/18:3) (Supplementary Figure S2D). TG (15:0/18:1/18:1) appeared in both the R4 versus R1 and R5 versus R1 comparisons.

### Analysis of lipid correlations

**Figure 6:**
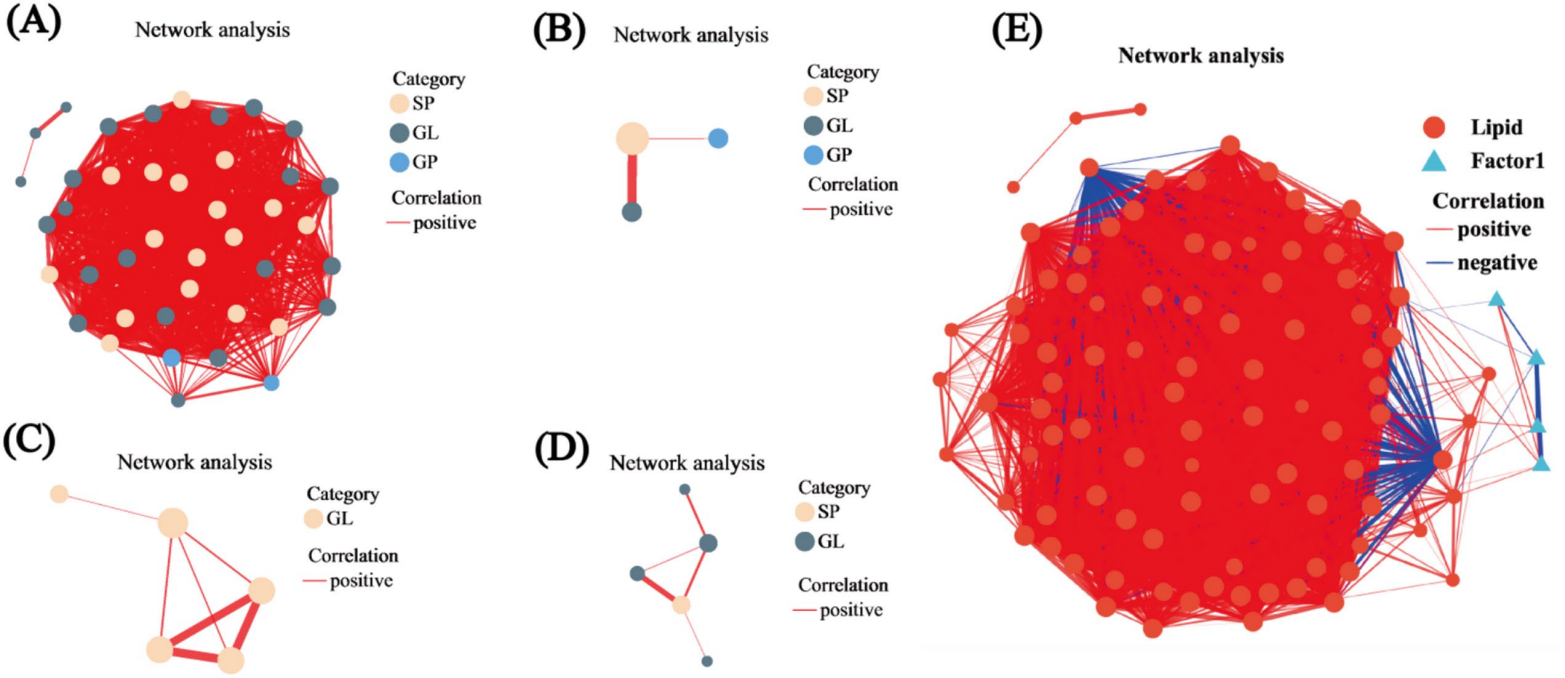
Univariate correlation network analysis. **(A)** Correlation network for R2 versus R1. **(B)** Correlation network for R3 versus R1. **(C)** Correlation network for R4 versus R1. **(D)** Correlation network for R5 versus R1. **(E)** Correlation network between lipid metabolites and environmental factors. In the figures, the size of the nodes indicates their degree, while different colors represent various classifications. The color of the connecting lines denotes the nature of the correlation, with red indicating positive correlation and blue indicating negative correlation. The thickness of the lines reflects the magnitude of the correlation coefficient.

We constructed networks and visualized them using network diagrams. The correlation network between R2 and R1 identified three types of lipids, namely, SP, GL, and GP, with respective counts of 17, 22, and 2. Within these lipid types, TG and Cer presented the highest counts at 18 and 16, representing 43.9 and 39.02% of the total lipid count, respectively. Notably, the clustering coefficient for TG (15:0/16:1/16:1) was 1, indicating complete connectivity among all adjacent nodes, while all Cer nodes had degree values exceeding 34 (Figure 6A). In the correlation network comparing R3 to R1, only three nodes were detected, corresponding to SP, GL, and GP lipids; the SP was TG (15:0/16:1/16:1) (Figure 6B). The R4 versus R1 network revealed 5 nodes, all of which were classified as GL lipids within the TG subclass: TG (16:0/16:0/16:0), TG (16:1/14:0/14:0), TG (15:0/18:1/18:1), TG (16:1/16:1/20:5), and TG (15:0/15:0/16:0). Notably, TG (16:0/16:0/16:0), TG (16:1/14:0/14:0), and TG (16:1/16:1/20:5) also exhibited clustering coefficients of 1 (Figure 6C). In the R5 versus R1 network, 5 nodes were identified, corresponding to SP and GL lipids, with three belonging to the TG subclass—TG (16:0/16:0/16:1), TG (15:0/18:1/18:1), and TG (16:1/18:3/18:3)—where TG (16:1/18:3/18:3) had a clustering coefficient of 1. TG (15:0/18:1/18:1) was also present, consistent with the R4 versus R1 comparison (Figure 6D). Analysis of the correlation network between lipid metabolites and environmental factors revealed that most nodes exhibited positive correlations, with two GP nodes showing negative correlations. Eleven lipids were found to be associated with environmental factors, including the TG, Cer, and LPC subclasses, with 8 TGs accounting for 72.72% of the total. The strongest associations were observed with S_POD, represented by TG (15:0/16:1/18:1), TG (18:1/17:1/18:1), TG (15:0/15:0/16:0), and TG (16:1/18:3/18:3) (Figure 6E).

### Analysis of microbial diversity

The analysis indicated a significant difference in bacterial community richness between the R4 and R5 groups (*p* < 0.05), while diversity and evenness showed no significant differences. In the case of fungi, differences in richness, diversity, and evenness were observed across the groups, but these differences were also not significant. Therefore, it is evident that flooding followed by drainage leads to significant changes in bacterial richness (Supplementary Table S9).

### Analysis of microbial community composition

The analysis revealed 1 shared species between R1 and R5; 2 species shared among R2, R3, R4, and R5; and 1 species shared among R1, R2, R4, and R5, resulting in a total of 10 shared fungal species across all treatment groups (Supplementary Figure S3B). The relative abundance analysis of the bacterial community revealed that the predominant phyla were *Actinobacteriota*, *Chloroflexi*, *Proteobacteria*, *Acidobacteriota*, and *Firmicutes*. The relative abundance of *Actinobacteriota* progressively increased from R1 to R5, with a relative abundance of 18.7% in R1 and reaching a maximum of 24.6% in R5. *Chloroflexi* accounted for 20.06, 18.90, 21.37, 19.85, and 19.79% of the total bacteria in R1 through R5, respectively, with R3 accounting for the highest proportion. In R5, *Proteobacteria* and *Acidobacteriota* exhibited relative abundances of 16 and 10.95%, respectively, while *Firmicutes* showed an abundance of 11.65%, averaging 11.33% overall. The relative abundances of the remaining species were all below 5% (Supplementary Figure S3C). In the fungal community relative abundance analysis, two primary phyla were identified: *Ascomycota* and *Rozellomycota*. *Ascomycota* consistently demonstrated the highest relative abundance across all treatment groups, with values of 48.42, 42.09, 44.58, 58.30, and 40.39%; the maximum was observed in R4 and minimum in R5, with an average value of 46.75%. *Rozellomycota* had an average abundance of 2.25%, with a relative abundance of 27.58% in R5. The remaining species did not exceed 25% relative abundance (Supplementary Figure S3D). The bacterial PCoA plot, showing PC1 and PC2 values of 36.46 and 32.21%, respectively, indicated significant overlap and similarity among the communities (*p* < 0.05) (Supplementary Figure S3E). The fungal PCoA plot revealed that PC1 and PC2 accounted for 61.09 and 15.20% of the variance, respectively, with overlapping communities, suggesting notable similarity in community composition (Supplementary Figure S3F).

### LEfSe multilevel species differential discrimination analysis

**Figure 7:**
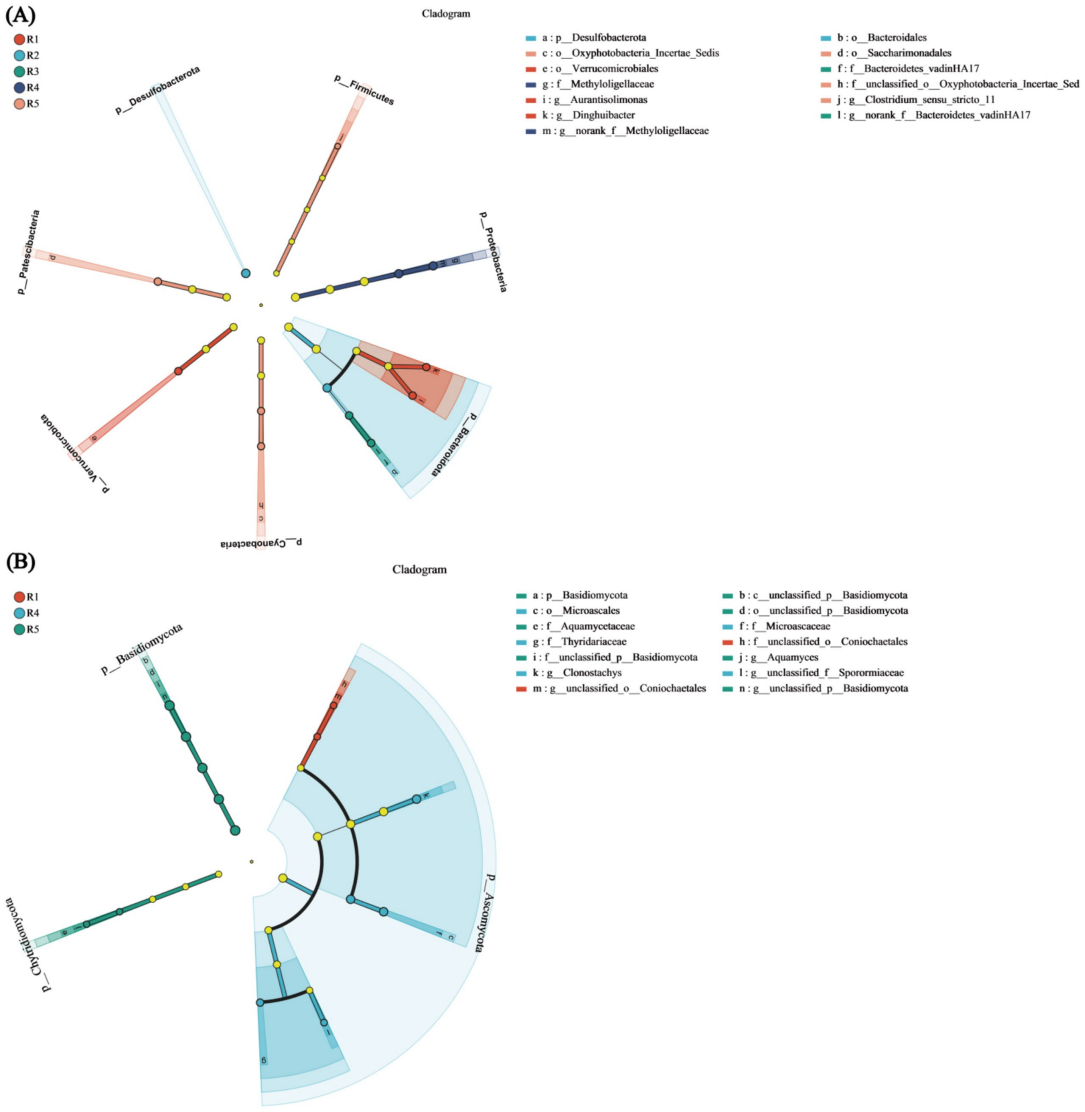
LEfSe multi-level species differential discrimination analysis. **(A)** Bacterial LEfSe multi-level species hierarchy tree diagram. **(B)** Fungal LEfSe multi-level species hierarchy tree diagram. Nodes in different colors indicate microbial taxa that are significantly enriched in the corresponding groups and have a notable impact on inter-group differences; light yellow nodes represent microbial taxa that show no significant differences across groups or do not significantly affect inter-group differences.

The bacterial LEfSe multilevel species hierarchy tree diagram identified eight differentially abundant species at the phylum level, including *Firmicutes*, *Proteobacteria*, *Bacteroidot*a, *Cyanobacteria*, *Chloroflexi*, *Verrucomicrobiot*a, *Patescibacteria*, and *Desulfobacterota*. Within the Firmicutes branch, *Clostridium_sensu_stricto_11* was significantly enriched (*p* < 0.05). For *Proteobacteria*, the two significantly enriched taxa were *Methyloligellaceae* and *Methyloligellaceae* (*p* < 0.05). In *Bacteroidota*, five significantly enriched species were identified, namely, *Bacteroidetes_vadinHA17*, *Bacteroidales*, and *Bacteroidetes_vadinHA17*, with both *Bacteroidales and Bacteroidetes_vadinHA17* belonging to the same category. Additionally, *Aurantisolimonas* and *Dinghuibacter* diverged from this category. The Cyanobacteria phylum also included two significantly enriched species: *Oxyphotobacteria_Incertae_Sedis* and *Oxyphotobacteria_Incertae_Sedis* (*p* < 0.05). Both Verrucomicrobiota and Patescibacteria contained one significantly enriched species each: *Verrucomicrobiales* and *Saccharimonadales*, respectively. Desulfobacterota had only one species without any other significantly enriched taxa (Figure 7A).

In the fungal LEfSe multilevel species hierarchy tree diagram, three differentially abundant taxa were identified: *Basidiomycota*, *Ascomycota*, and *Chytridiomycota*. Within *Basidiomycota*, four species were significantly enriched from the phylum to the genus level: *Class Basidiomycota*, *Order Basidiomycota*, *Family Basidiomycota*, and *Genus Basidiomycota*. In Ascomycota, there were no significantly enriched species at the phylum or class level; however, at the order level, *Microascaceae* and *Thyridariaceae* were significantly enriched (*p* < 0.05). At the family level, two significantly enriched species were identified (*p* < 0.05), namely, *unclassified_o_Coniochaetales* and *Microascales*. At the genus level, three significantly enriched species were found: *unclassified_o_Coniochaetales*, *Clonostachys*, and *unclassified_f_Sporormiaceae*. Chytridiomycota showed significant enrichment exclusively at the family and genus levels, specifically in *Aquamyces* and *Aquamycetaceae* (*p* < 0.05) (Figure 7B). Analysis of the LEfSe multilevel species hierarchy tree diagram revealed that *Bacteroidota* (bacteria) and *Ascomycota* (fungi) were the most abundant and taxonomically rich groups in both comparisons, suggesting their potential ecological importance in the soil environment during the water recession stage.

### Correlation analysis

RDA of bacterial taxa and environmental factors was conducted at the phylum level; the results revealed that RDA1 accounted for 17.65% and RDA2 accounted for 5.23%, these values explained an small percentage of variance. The environmental factor with the greatest influence on bacteria was S_CAT, followed by S_POD, and the least impactful factor was S_PPO, with all three factors exhibiting positive correlations. These factors were found in the first quadrant and were therefore negatively correlated with RDA1 and positively correlated with RDA2 (Supplementary Figure S4A). In the analysis of fungal taxa and environmental factors, RDA1 represented 42.24%, and RDA2 represented 1.17%. The order of environmental factors impacting fungi, from greatest to least, was S_CAT, S_PPO, and S_POD, with all exhibiting negative correlations among themselves. S_CAT was in the first quadrant, negatively correlated with RDA1 and positively correlated with RDA2, while S_PPO was in the fourth quadrant, positively correlated with RDA1 and negatively correlated with RDA2. S_POD was also in the first quadrant and was negatively correlated with both RDA1 and RDA2 (Supplementary Figure S4B). The correlations between bacteria and metabolites were further analyzed. PA (10:0/PGDI) was significantly negatively correlated with *Zixibecteria*, *Campilobacterota*, *Dadabacteria*, *Methylomirabilota*, and *FW113* but significantly positively correlated with *Spirochaetota*, *Deinococcota*, and *Firmicutes* (*p* < 0.05). PG(TXB2/a-25:0) was significantly positively correlated with *Bacteroidota* and Firmicutes (*p* < 0.05). MG(a-13:0/0:0/0:0)[rac] was significantly positively correlated with *Cyanobacteria* (*p* < 0.05). Cer(d17:1/PGD2) was significantly positively correlated with *Methylomirabilota* but negatively correlated with *Spirochaetota* (*p* < 0.05), *Deinococcota*, *Firmicutes*, and *Hydrogenedentes*. LysoPA(a-25:0/0:0) was significantly positively correlated with *Campilobacterota* and negatively correlated with *Margulisbacteria* (*p* < 0.05), *Halanaerobiaeota*, and *Hydrogenedentes*. Cer(d17:1/PGE2) had no significantly positively correlated metabolites, while Cer(d17:1/PGD2) exhibited significant negative correlations with several taxa (*p* < 0.05) (Supplementary Figure S4C). Among the fungal metabolites, Cer(d17:1/PGD2) was significantly negatively correlated with *Kickxellomycota* and *Rozellomycota* (*p* < 0.05), while Cer(d17:1/PGE2) was negatively correlated with *Kickxellomycota*, *Calcarisporiellomycota*, and *Blastocladiomycota*. LysoPA(a-25:0/0:0) was significantly negatively correlated with *Kickxellomycota* and *Calcarisporiellomycota* (*p* < 0.05). Neither MG(a-13:0/0:0/0:0)[rac] nor PG(TXB2/a-25:0) was significantly correlated with any metabolites (*p* < 0.05). PA(10:0/PGD1) was positively correlated with Rozellomycota (Supplementary Figure S4D).

### Correlation network analysis

**Figure 8:**
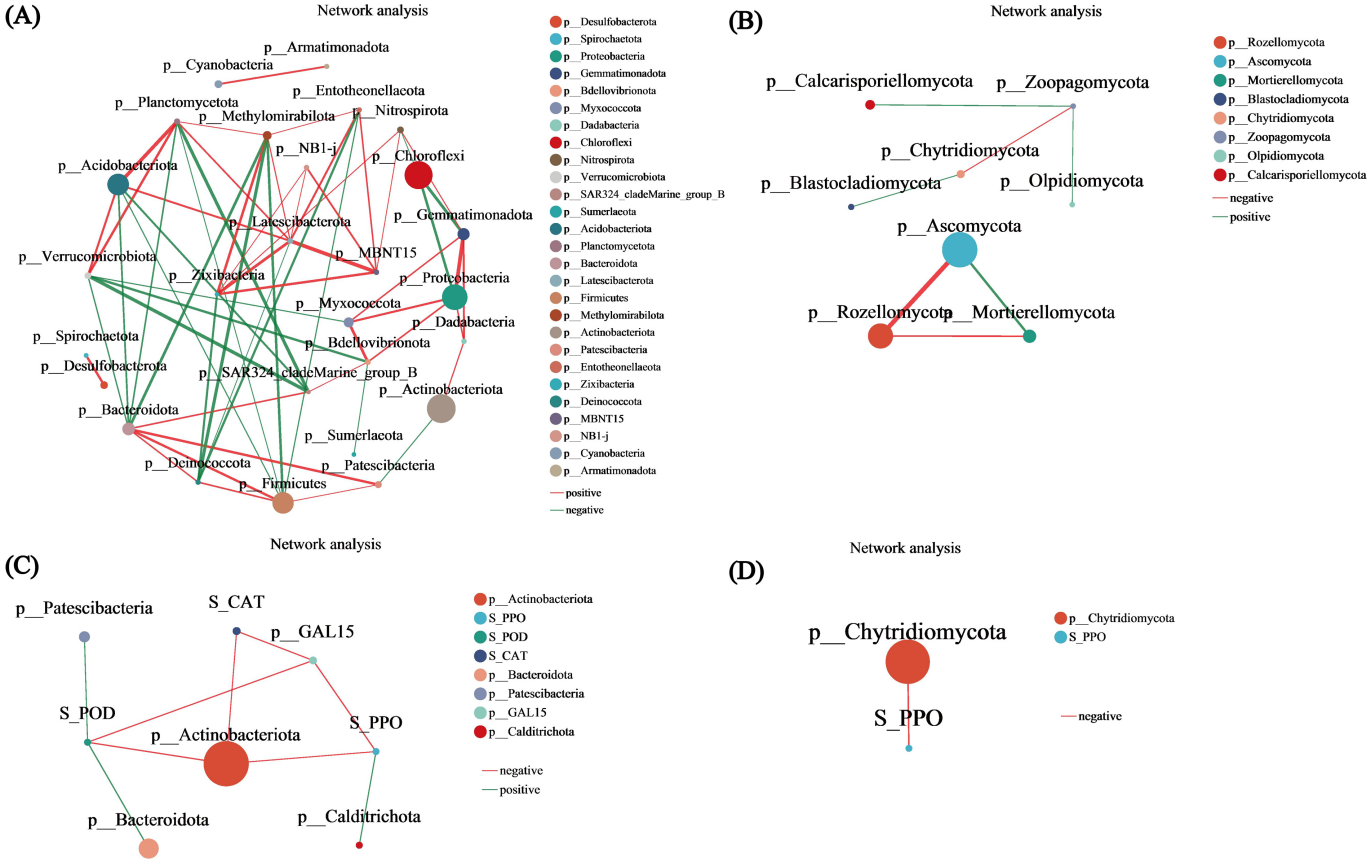
Correlation network analysis. **(A)** Bacterial univariate network diagram. **(B)** Fungal univariate network diagram. **(C)** Bacterial bivariate network diagram. **(D)** Fungal bivariate network diagram. The bivariate correlation network diagram primarily illustrates the correlation between species at a specific taxonomic level and environmental factors within the samples. The diagram displays species information with correlation coefficients of absolute values greater than or equal to 0.6 and *p* < 0.05. The color of the lines indicates the type of correlation: red represents positive correlations, while green indicates negative correlations. The thickness of the lines corresponds to the strength of the correlation; thicker lines signify stronger correlations between species, and a greater number of lines indicates closer connections between that species and others.

Bacterial unifactorial network diagram analysis revealed associations among bacterial species at 27 phylum levels. Among these, five species exhibited relative abundances exceeding 6,000: *Actinobacteriota*, *Chloroflexi*, *Proteobacteria*, *Acidobacteriota*, and *Firmicutes*, with *Actinobacteriota* having the highest abundance of 113,633. *Latescibacterota* and *Bacteroidota* displayed the most associations with other species, with a total of 8 associations. Notably, the clustering coefficient of *NB1-j* was 1, indicating that its nodes were fully connected with neighboring nodes. An analysis of the three centrality coefficients of all nodes revealed that *Bacteroidota* consistently ranked among the top five across all three centrality metrics, highlighting its significance in the network (*p* < 0.05) (Figure 8A). Similarly, the fungal unifactorial network diagram revealed associations among fungal species in 8 phyla. Among these, *Ascomycota* and *Rozellomycota* presented higher abundances. *Mortierellomycota*, *Ascomycota*, and *Rozellomycota* were independently associated, with clustering coefficients of 1, suggesting that these three species are completely interconnected. *Zoopagomycota* exhibited the highest number of associations with other species, totaling 3, and also had the highest values across all three centrality coefficients (Figure 8B). The correlation network analysis between bacterial metabolites and environmental factors revealed that most node-to-node interactions were negatively correlated. However, S_POD was positively correlated with *Patescibacteria* and *Bacteroidota*, while S_PPO was positively correlated with *Calditrichota*. The analysis indicated that *Actinobacteriota* had the highest abundance and was influenced by three environmental factors. S_CAT affected both *Actinobacteriota* and *p__GAL15* (Figure 8C). In contrast, the fungal metabolite–environment factor correlation network showed that only one environmental factor influenced one metabolite: S_PPO negatively affected *Chytridiomycota*, although its clustering coefficient was 0, indicating minimal association between them (Figure 8D).

## Discussion

The oxidation potential of the soil increased with the duration of drainage (Figure 1D), corroborating earlier research (Harpenslager et al., 2024; Mikutta et al., 2024). These results indicate that flooding and subsequent drainage significantly impact the physiological and biochemical characteristics of the soil. The PCA of four comparison groups revealed that R2-R1 and R5-R1 exhibited significant overlap (*p* < 0.05), indicating a certain degree of similarity (Figures 2A,D). In contrast, R3-R1 and R4-R1 were more distantly spaced, indicating differences (Figures 2B,C). Partial least squares discriminant analysis (PLS-DA) demonstrated clear separation among the four groups, underscoring the strong classification effect of the metabolites (Figures 2E–H). Metabolite analysis showed that in the R2-R1, R3-R1, and R4-R1 comparison groups, the number of downregulated metabolites exceeded the number of upregulated metabolites, with R3-R1 displaying the highest number of differentially expressed metabolites. Conversely, R5-R1 had more upregulated metabolites (Figure 3A). The Venn diagram of metabolites indicated that R5-R1 possessed the highest number of unique metabolites (Figure 3B), suggesting that the soil microorganisms experienced metabolite profile shifts under flooding and drainage conditions, followed by redox reactions over time. This aligns with previous findings from metabolomic analyses of flooded and postflooding grape seedlings (Peng et al., 2023). Additionally, the four comparison groups revealed the presence of diverse organic heterocyclic compounds, lipids and lipid-like molecules, organic acids and their derivatives, benzoic acids, organic oxidants, and phenolic compounds (Figures 3C–F), consistent with earlier studies (Wang et al., 2023).

The distribution aligns with previous findings on lipid distributions under salt stress (Xue et al., 2024). Additionally, the radar chart of unsaturated lipid contents revealed the prevalence of 16:0, 16:1, 18:0, 18:1, and 18:2 lipids, consistent with prior research (Couvillion et al., 2023). The lipid metabolites were predominantly classified into four categories: GL, SP, GP, and FA (Figure 5A). In the lipid classification summary table, the most abundant lipid subclasses were TG from the GL class and Cer from the SP class (Figure 5B), corroborating findings from Pérez-Cova et al. (2022) and Xu et al. (2024). Among the comparison groups, R2-R1 exhibited the highest number of lipid metabolites, while the other three groups each had five (Supplementary Figure S1A). Unique lipid metabolites were also most abundant in the R2-R1 group, suggesting that lipid redox reactions stabilized over time, aligning with previous studies (Couvillion et al., 2023). Further validation through lipid differential classification analysis revealed the highest number of differential lipid species in the R2-R1 group, with TG lipids present in all comparison groups (Supplementary Figures S2A–D). Correlation network analysis indicated that R2-R1 had the most lipids, with TG (15:0/16:1/16:1) exhibiting a clustering coefficient of 1, indicating complete connectivity among adjacent nodes (Figure 6A). TG lipids (a subclass of GLs) were present in all groups (Figures 6B–D). Additionally, 11 lipid types were correlated with environmental factors, with TG lipids constituting 72.72% of the total lipids (Figure 6E). In previous studies, it has been found that the utilization efficiency of microbial carbon sources decreases, making it easier to accumulate neutral energy storage lipids such as TAG. And after the restoration of oxidative conditions, plant roots and microorganisms initiate β oxidation to decompose TAG for energy supply (Sun et al., 2018; Dourou et al., 2018). These results highlight significant redox reactions in root soil lipids under flooding and drainage stress, confirming that TG lipids are major reductive lipids, consistent with previous findings (Chialva et al., 2020). This study found that the dominant bacterial communities Actinobacteriota and TG lipids undergo changes during rehydration. Previous studies have shown that bacterial TG biosynthesis is mainly limited to the phylum Actinobacteria (Alvarez and Steinbüchel, 2002; Zhang et al., 2024), and fatty acids can enter the TCA cycle through ß oxidation or be resynthesized by fatty acid synthase. Therefore, we believe that Actinobacteriota is correlated with TG lipids during rehydration. Meanwhile, previous studies have shown that microbial lipid metabolism (including TG) is closely related to Eh values. Through simulated oxidation/reduction alternation experiments, it was revealed how the red oxygen cycle controls Fe mineral dissolution and organic carbon release. Under lower Eh conditions, organic carbon tends to passively release and accumulate through reduction. Although TG lipids were not directly analyzed, it was emphasized that microbial organic carbon metabolism (including neutral lipid branch metabolism such as TG) may be regulated in a reducing environment (Schulz et al., 2024). The study emphasizes that the availability of oxygen determines the accumulation of Fe^2+^, microbial biomass, and degradability of C, with the central role of Fe as an electron acceptor mediating SOC mineralization (Li et al., 2021). In this study, it was found that TG lipids are correlated with physiological and biochemical characteristics, and changes in Eh can be found to regulate the synthesis and decomposition pathways of TG.

This study examined bacterial and fungal community richness, diversity, and evenness across various treatment groups, identifying significant differences in bacterial richness between R4 and R5 (*p* < 0.05) (Supplementary Table S3), aligning with earlier research (Zhu et al., 2022; Gao et al., 2023). The bacterial community was primarily composed of *Actinobacteriota*, *Chloroflexi*, *Proteobacteria*, and *Acidobacteriota* (Supplementary Figure S3C), aligning with earlier research on rice rhizosphere bacteria under various water management conditions (Chialva et al., 2020) and confirming the predominant bacterial composition under drought and flooding stress in corn and wheat (Gao et al., 2023; Francioli et al., 2021). These findings are consistent with prior studies examining rice rhizosphere microbiota under diverse irrigation regimes (Chialva et al., 2020) and further validate the characteristic bacterial assemblages observed in cereal crops subjected to drought and flooding stress (Gao et al., 2023; Francioli et al., 2021).

For fungi, *Ascomycota* and *Rozellomycota* were the main groups identified under flooding and drainage stress (Supplementary Figure S3D), corroborating previous studies (Li et al., 2020). These microbial communities are consistent with those mentioned in previous studies that can promote plant growth (Santoyo et al., 2016). PCoA revealed high similarity in community composition among the five groups of bacteria and fungi (Supplementary Figures S3E,F), supporting earlier studies (Chialva et al., 2020). LEfSe analysis revealed that for bacteria, *Bacteroidota* was significantly enriched in R2, R3, and R4 (*p* < 0.05), while for fungi, *Ascomycota* was predominantly enriched in R1 and R4, particularly in R4 (Francioli et al., 2021). RDA revealed that the environmental factors S_CAT, S_POD, and S_PPO were positively correlated with bacterial communities but negatively correlated with fungal communities, confirming that external water stress affected bacterial richness but not fungal richness. Previous research has indicated that increased soil enzyme activity is linked to increased microbial biomass due to precipitation (Yang et al., 2017), while changes in pH due to variations in water levels affect soil enzyme activity (Xu et al., 2023), supporting our findings. In the single-factor correlation network analysis, *Actinobacteriota*, *Chloroflexi*, *Proteobacteria*, and *Acidobacteriota* had the highest relative abundances among bacteria, with *Actinobacteriota* being the most abundant and *Bacteroidota* having the most associations (Supplementary Figures S4A,B). Among fungi, *Ascomycota* and *Rozellomycota* were relatively abundant. Previous studies have shown that Ascomycota and Rozellomycota play roles in promoting organic matter decomposition, secreting secondary metabolites, and supporting autotrophic processes (Yang et al., 2025). Correlation analysis revealed that *Actinobacteriota* were influenced by three environmental factors (Supplementary Figure S4C), while among fungi, *Chytridiomycota* were impacted by soil polyphenol oxidase (Supplementary Figure S4D). The significant correlation observed between fungal diversity, community composition, and phenol oxidase activity in previous studies aligns well with the results obtained in this study (Toberman et al., 2008).

This study examined the dynamic changes in soil microbial community structure, function, and metabolome during flooding and drainage. The findings indicated decreases in S-PPO, S-POD, and S-CAT with prolonged drainage, along with increases in soil redox potential (Eh-mV) and POD over time. The composition and changes in major soil metabolites, lipids, fungi, and bacteria were also analyzed, providing a framework for further understanding the adaptation of rice soil ecosystems under adverse conditions. Due to the rapid changes in soil redox status caused by flooding and water withdrawal, the microbial community around the roots is affected, which in turn affects the nutrient absorption and resistance expression of rice. Additionally, the accumulation of nitrite and the entry of oxygen during drainage promote denitrification reactions and increase carbon dioxide emissions. Therefore, understanding the microbial metabolic network reconstruction induced by flooding and water withdrawal is of great guiding significance for optimizing agricultural management and mitigating greenhouse gas emissions.

## Conclusion

In this study, flooding and subsequent drainage significantly influenced the composition of soil microbial communities and lipid profiles. With prolonged drainage, the activities of key soil enzymes—including polyphenol oxidase, peroxidase, and catalase—gradually declined, whereas the soil redox potential increased over time. Redox changes in lipids were associated primarily with triglycerides, which, based on their dynamic behavior during drainage, were rapidly degraded and involved in oxidative energy supply. Notably, the most substantial lipid reduction occurred on the second day post-drainage. Drainage also markedly altered bacterial community structure, and environmental factors played a regulatory role in mediating the correlation between bacterial communities and lipid profiles. These findings suggest a coordinated interaction among microbial communities, lipid metabolites, and environmental variables. Moreover, the observed temporal shifts in microbial composition and lipid metabolism during drainage highlight their integrated response to changing hydrological conditions, which may contribute to enhanced soil ecosystem stability and adaptive resilience under external stressors.

## Data Availability

Sequencing data for this project has been deposited in the Genome Sequence Archive of the Big Data Center at the Beijing Institute of Genomics (BIG), Chinese Academy of Sciences (http://bigd.big.ac.cn), the archive number is CRA022589.

## Footnotes

^1^ http://www.hmdb.ca/

^2^ https://metlin.scripps.edu/

^3^ https://cloud.majorbio.com

^4^ https://www.kegg.jp/kegg/pathway.html

## Glossary

S-PPO: Soil polyphenol oxidase
S-POD: Soil peroxidase
S-CAT: Soil catalase
Eh-mV: Soil redox potential
SFAs: Saturated fatty acyls
MUFAs: Monounsaturated fatty acyls
PUFAs: Polyunsaturated fatty acyls
ODD: Odd-chain fatty acyls
GLs: Glycerolipids
GPs: Glycerophospholipids
SPs: Sphingolipids
DGs: Diacylglycerols
MG: Monostearin
MGDGs: Monogalactosyl diacylglycerols
TGs: Triacylglycerols
PC: Diacylglycerophosphocholine
LPC: Lysophosphatidylcholine
PE: Diacylglycerophosphoethanolamine
PEt: Phosphatidylethanolamine
PI: Diacylglycerophosphoinositol
Cer: Ceramides
SM: Sphingomyelin
Sph: Sphingosine
ST: Sterol
WE: Wax esters
Co: Cholesteryl esters
FA: Fatty acid
