# Supplementary material for: Rapid reshaping of the soil microbiome and metabolome during short-term flooding and draining in rice
Source: Front Microbiol. 2025 Sep 2;16:1632744. doi: 10.3389/fmicb.2025.1632744 (PMC12436361; doi:10.3389/fmicb.2025.1632744)
Supplement: Supplementary file 1 [file Table_1.DOCX]

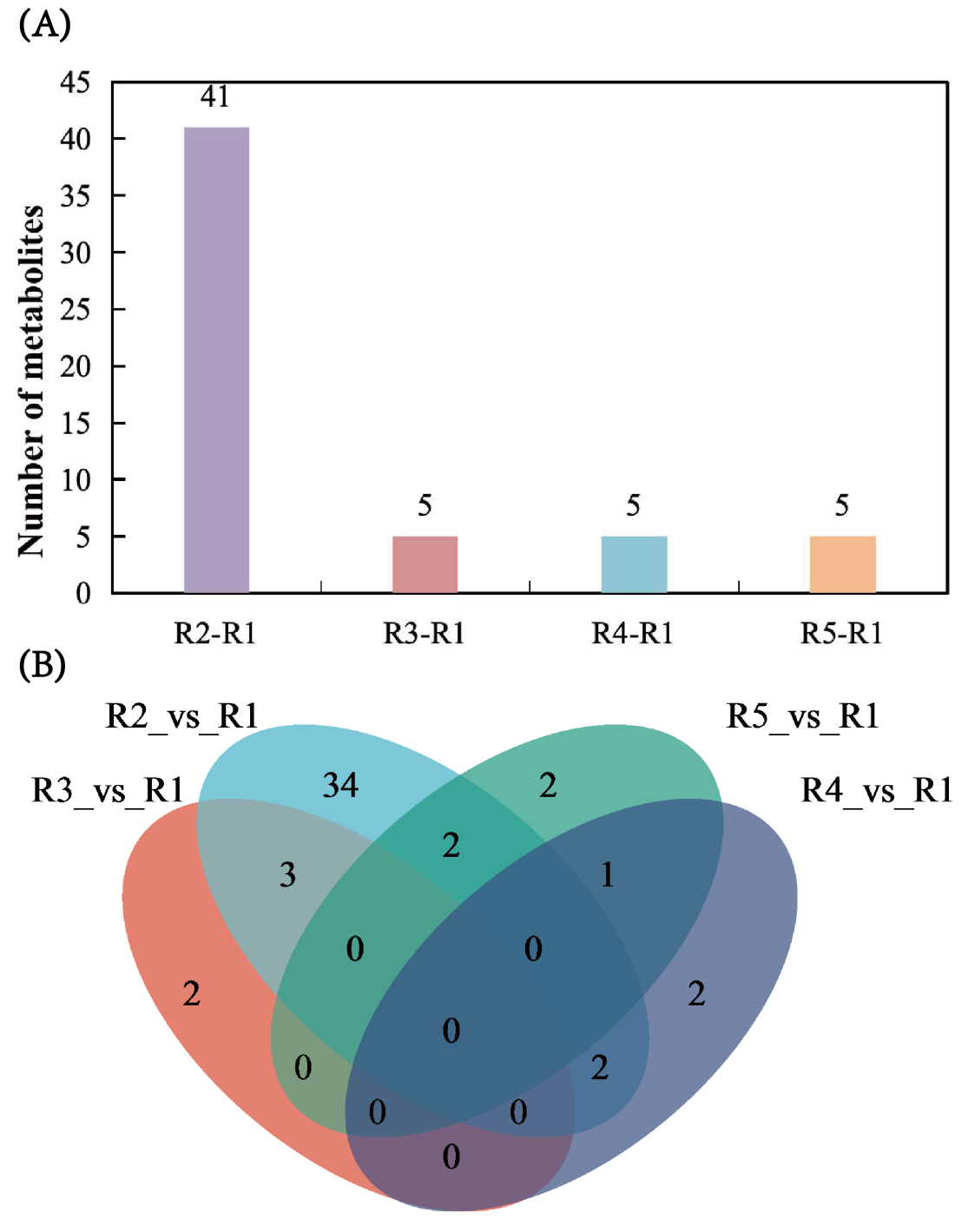


**Figure S1.** Analysis of Lipid Metabolites. (A) Bar chart illustrating metabolite counts across different comparison groups. The chart shows the number of metabolites in each metabolite set. (B) Venn diagram depicting the number of shared metabolites among the comparison groups. The overlapping sections indicate the number of metabolites common to multiple sets, while the non-overlapping sections represent the unique metabolites specific to each set. The numbers correspond to the respective counts of metabolites.
