# Supplementary material for: Rapid reshaping of the soil microbiome and metabolome during short-term flooding and draining in rice
Source: Front Microbiol. 2025 Sep 2;16:1632744. doi: 10.3389/fmicb.2025.1632744 (PMC12436361; doi:10.3389/fmicb.2025.1632744)
Supplement: Supplementary file 2 [file Table_2.DOCX]

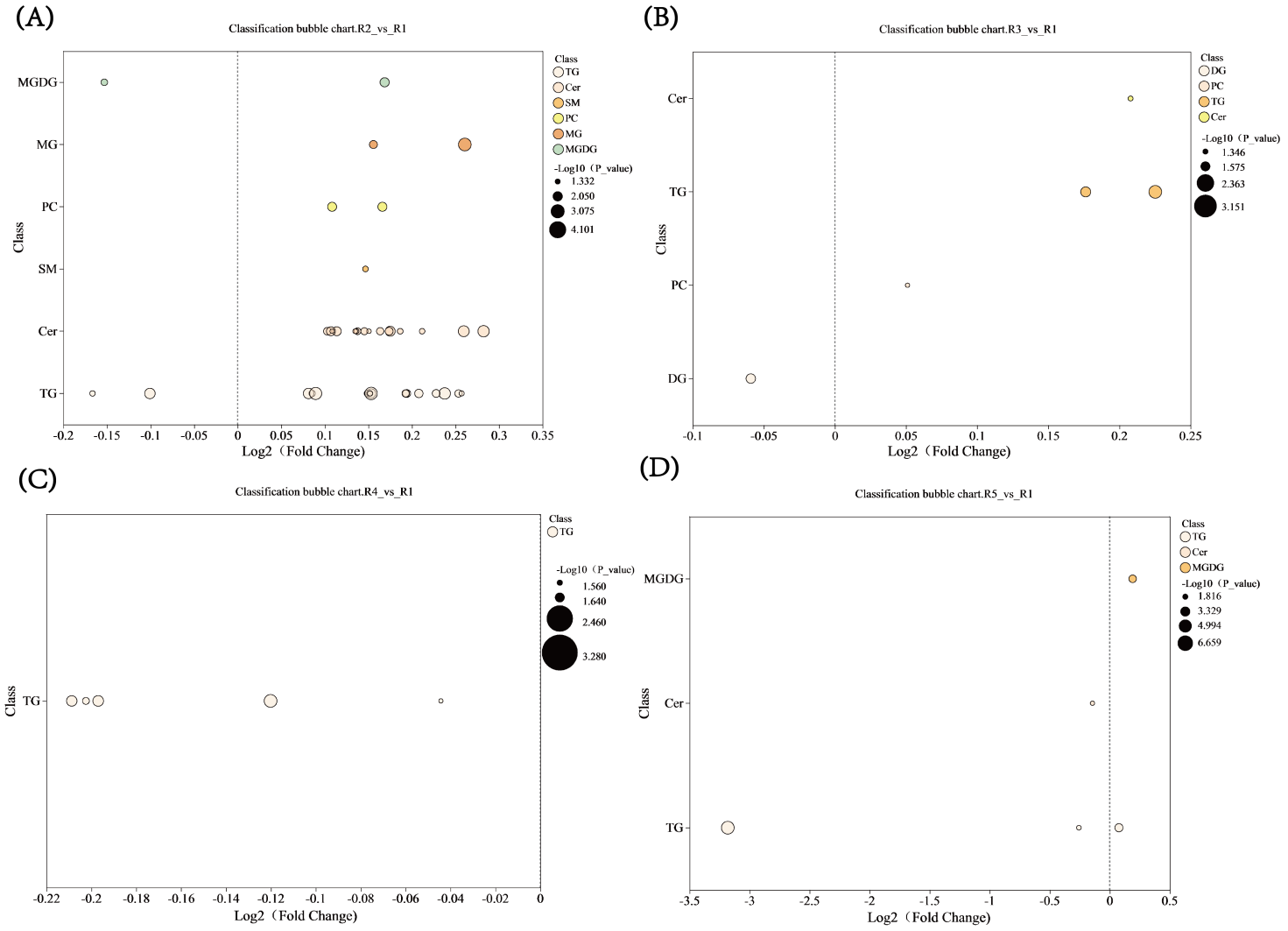


**Figure S2.** Analysis of Differential Lipid Classification. (A) Scatter plot of differential lipids between R2 and R1. (B) Scatter plot illustrating the differential lipids between R3 and R1. (C) Scatter plot illustrating the differential lipids between R4 and R1. (D) Scatter plot of differential lipids between R5 and R1. In the figures, each distinct dot represents a metabolite, with the x-axis indicating the fold change in expression between the two groups, expressed as Log2(fold change). The y-axis displays lipid subclass classifications, with different colors denoting each classification.
