# Supplementary material for: Rapid reshaping of the soil microbiome and metabolome during short-term flooding and draining in rice
Source: Front Microbiol. 2025 Sep 2;16:1632744. doi: 10.3389/fmicb.2025.1632744 (PMC12436361; doi:10.3389/fmicb.2025.1632744)
Supplement: Supplementary file 3 [file Table_3.DOCX]

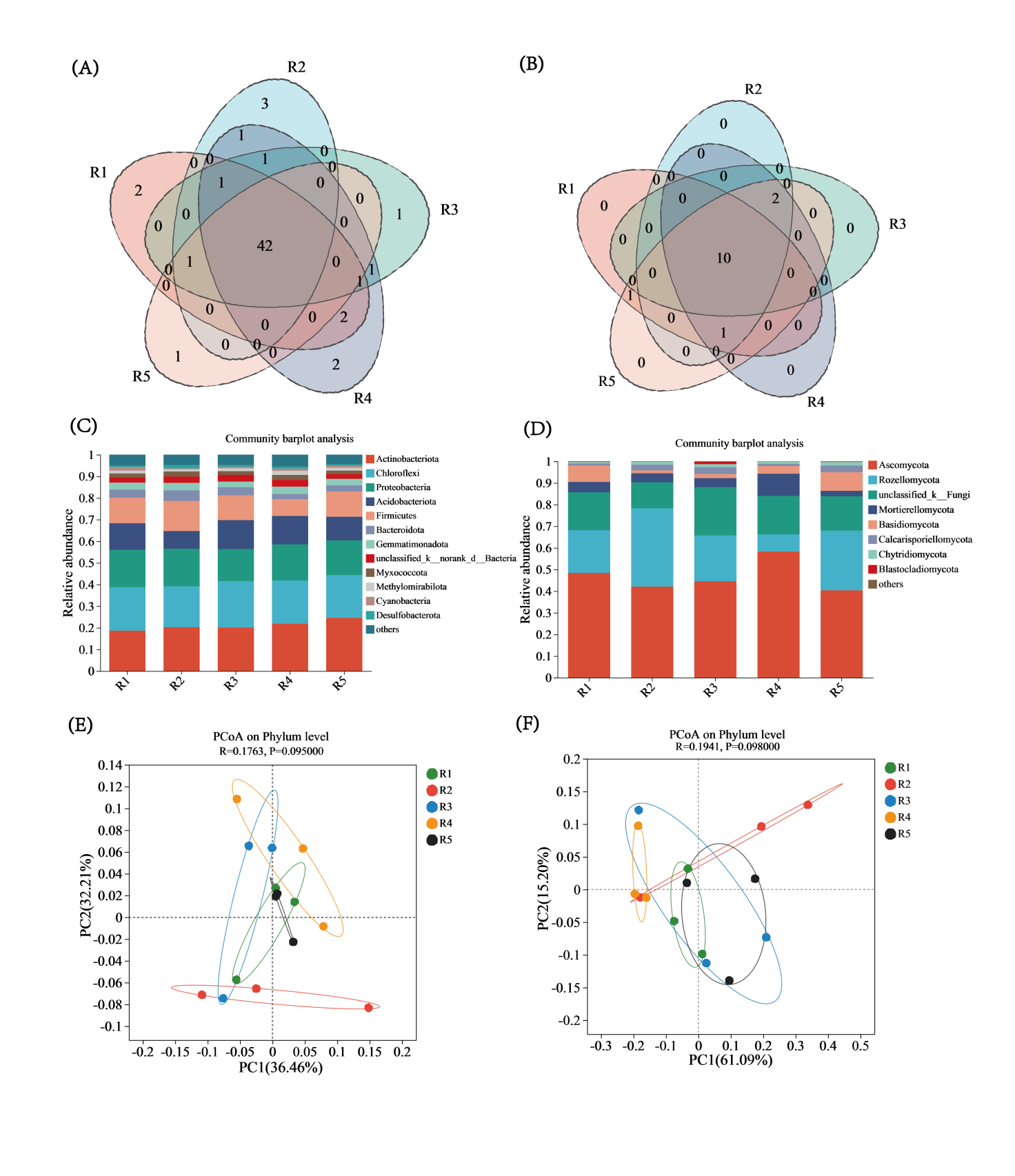


**Figure S3.** Analysis of Community Composition. (A) Bacterial Venn diagram. (B) Fungal Venn diagram. Different colors represent different groups (or samples), with overlapping areas indicating species shared among multiple groups (or samples), while non-overlapping areas indicate species unique to each group (or sample). The numbers indicate the respective species counts. (C) Bar chart of bacterial relative abundance. (D) Bar chart of fungal relative abundance. The bars are color-coded to represent various species, with their length indicating the proportion of each species. Beta diversity analysis. (E) Bacterial PCoA plot. (F) Fungal PCoA plot. The x- and y-axes denote two principal coordinate components, with the percentages showing each component's contribution to the variation in sample composition.
