# Supplementary material for: Rapid reshaping of the soil microbiome and metabolome during short-term flooding and draining in rice
Source: Front Microbiol. 2025 Sep 2;16:1632744. doi: 10.3389/fmicb.2025.1632744 (PMC12436361; doi:10.3389/fmicb.2025.1632744)
Supplement: Supplementary file 4 [file Table_4.DOCX]

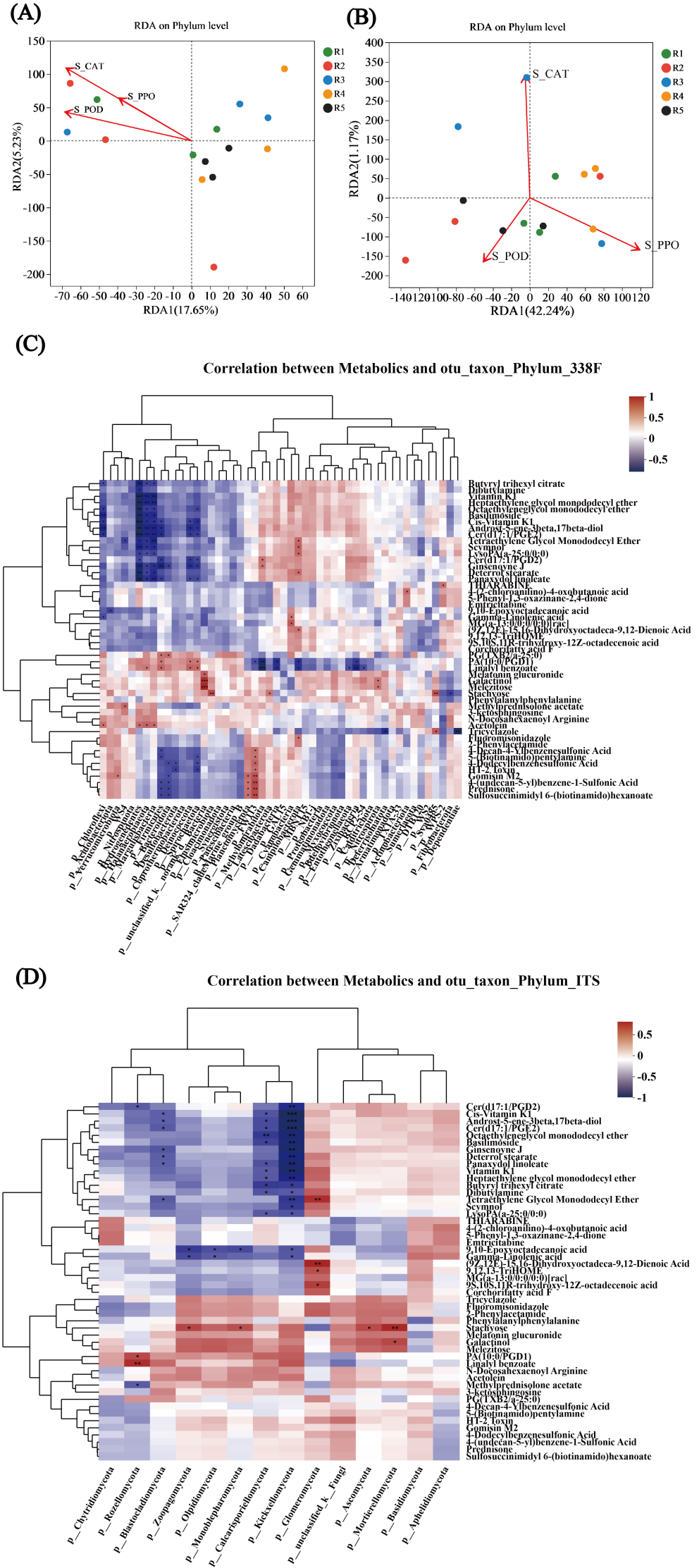


**Figure S4.** RDA Results. (A) RDA scatter plot of bacteria. (B) RDA scatter plot of fungi. In the figure, distinct colors or shapes denote sample groups subjected to different environments or conditions. Light blue inverted triangles denote species, and red arrows indicate quantitative environmental factors. The length of the environmental factor arrows indicates the magnitude of their influence (explanatory power) on the distribution of species data among different sample groups. The correlation between environmental factors is indicated by the angles between arrows: acute angles suggest positive correlations, obtuse angles indicate negative correlations, and right angles imply no correlation.
