# Supplementary material for: Rapid reshaping of the soil microbiome and metabolome during short-term flooding and draining in rice
Source: Front Microbiol. 2025 Sep 2;16:1632744. doi: 10.3389/fmicb.2025.1632744 (PMC12436361; doi:10.3389/fmicb.2025.1632744)
Supplement: Supplementary file 5 [file Table_5.DOCX]

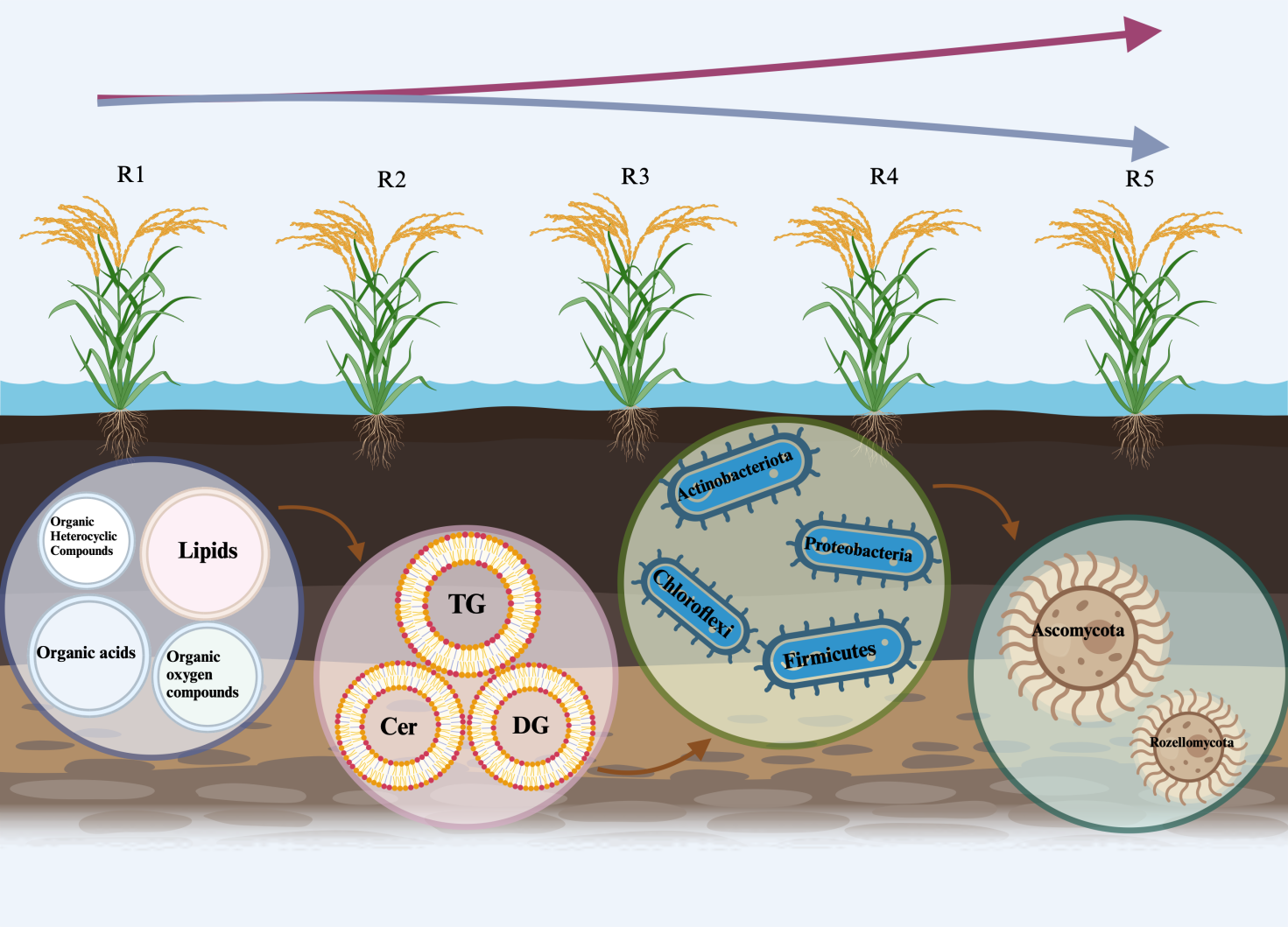
**Figure S5.** Conditions of soil microbes and the metabolome during the flooding-drainage process of rice.
