# Supplementary material for: Rapid reshaping of the soil microbiome and metabolome during short-term flooding and draining in rice
Source: Front Microbiol. 2025 Sep 2;16:1632744. doi: 10.3389/fmicb.2025.1632744 (PMC12436361; doi:10.3389/fmicb.2025.1632744)
Supplement: Supplementary file 14 [file Table_14.DOCX]

Table S9. Richness, Diversity, and Evenness of Bacterial and Fungal Communities Across Treatment Groups.

|  | **Bacteria** | | | **Fungi** | | |
| --- | --- | --- | --- | --- | --- | --- |
|  | **Chao** | **Shannon** | **Pielou_e** | **Chao** | **Shannon** | **Pielou_e** |
| R1 | 42.33ab | 2.29a | 0.61a | 10.33a | 1.33a | 0.57a |
| R2 | 43.42ab | 2.26a | 0.60a | 11a | 1.23a | 0.51a |
| R3 | 43ab | 2.23a | 0.59a | 10.67a | 1.36a | 0.57a |
| R4 | 44.73a | 2.28a | 0.60a | 11a | 1.40a | 0.58a |
| R5 | 40b | 2.20a | 0.60a | 11a | 1.35a | 0.56a |
